# Development of a prognostic risk model for clear cell renal cell carcinoma by systematic evaluation of DNA methylation markers

**DOI:** 10.1186/s13148-021-01084-8

**Published:** 2021-05-04

**Authors:** S. C. Joosten, S. N. O. Odeh, A. Koch, N. Buekers, M. J. B. Aarts, M. M. L. L. Baldewijns, L. Van Neste, S. van Kuijk, L. J. Schouten, P. A. van den Brandt, V. C. Tjan-Heijnen, M. van Engeland, K. M. Smits

**Affiliations:** 1 Department of Pathology, GROW – School for Oncology and Developmental Biology, Maastricht University Medical Center, P.O. Box 5800, 6202 AZ Maastricht, The Netherlands; 2 Department of Medical Oncology, GROW – School for Oncology and Developmental Biology, Maastricht University Medical Center, Maastricht, The Netherlands; 3 Department of Pathology, University Hospitals Leuven, Leuven, Belgium; 4 Department of Clinical Epidemiology and Medical Technology Assessment, GROW – School for Oncology and Developmental Biology, Maastricht University Medical Center, Maastricht, The Netherlands; 5 Department of Epidemiology, GROW – School for Oncology and Developmental Biology, Maastricht University Medical Center, Maastricht, The Netherlands

**Keywords:** Clear cell renal cell carcinoma (ccRCC), Prognostic model, DNA methylation biomarkers, DNA methylation location, TCGA data

## Abstract

**Background:**

Current risk models for renal cell carcinoma (RCC) based on clinicopathological factors are sub-optimal in accurately identifying high-risk patients. Here, we perform a head-to-head comparison of previously published DNA methylation markers and propose a potential prognostic model for clear cell RCC (ccRCC).

**Patients and methods:**

Promoter methylation of *PCDH8, BNC1, SCUBE3, GREM1, LAD1, NEFH, RASSF1A, GATA5, SFRP1, CDO1, and NEURL* was determined by nested methylation-specific PCR. To identify clinically relevant methylated regions, The Cancer Genome Atlas (TCGA) was used to guide primer design. Formalin-fixed paraffin-embedded (FFPE) tissue samples from 336 non-metastatic ccRCC patients from the prospective Netherlands Cohort Study (NLCS) were used to develop a Cox proportional hazards model using stepwise backward elimination and bootstrapping to correct for optimism. For validation purposes, FFPE ccRCC tissue of 64 patients from the University Hospitals Leuven and a series of 232 cases from The Cancer Genome Atlas (TCGA) were used.

**Results:**

Methylation of *GREM1, GATA5, LAD1, NEFH, NEURL*, and *SFRP1* was associated with poor ccRCC-specific survival, independent of age, sex, tumor size, TNM stage or tumor grade. Moreover, the association between *GREM1, NEFH,* and *NEURL* methylation and outcome was shown to be dependent on the genomic region. A prognostic biomarker model containing *GREM1, GATA5, LAD1, NEFH* and *NEURL* methylation in combination with clinicopathological characteristics, performed better compared to the model with clinicopathological characteristics only (clinical model), in both the NLCS and the validation population with a c-statistic of 0.71 versus 0.65 and a c-statistic of 0.95 versus 0.86 consecutively. However, the biomarker model had limited added prognostic value in the TCGA series with a c-statistic of 0.76 versus 0.75 for the clinical model.

**Conclusion:**

In this study we performed a head-to-head comparison of potential prognostic methylation markers for ccRCC using a novel approach to guide primers design which utilizes the optimal location for measuring DNA methylation. Using this approach, we identified five methylation markers that potentially show prognostic value in addition to currently known clinicopathological factors.

**Supplementary Information:**

The online version contains supplementary material available at 10.1186/s13148-021-01084-8.

## Highlights

- This work represents a head to head comparison of promising published DNA methylation biomarkers for ccRCC.
- TCGA data was used to select optimal DNA methylation location to guide primer design.
- We propose a new prognostic model for ccRCC adding five DNA methylation markers to the standard clinicopathological factors.
- Preliminary results suggest added clinical value and merit further validation in larger cohort studies.

## Introduction

Renal cell carcinoma (RCC) is the most common malignant neoplasm of the kidney and accounts for 2–3% of the total human cancer burden [1]. Clear cell RCC (ccRCC) is the most prevalent histopathological subtype, comprising 80–90% of all RCC cases [2]. Patients with non-metastatic ccRCC are treated with curative intent by (partial) nephrectomy. The clinical course after surgery is however erratic, as approximately 30% of these patients still develop metastases during follow-up and around ~ 10% die of disease progression within five years after surgery [3]. Currently, it is difficult to recognize these high-risk patients upon initial diagnosis. Despite the fact that current clinical characteristics or prognostic models, such as the TNM staging system, Fuhrman grading, the Stage, Size, Grade, and Necrosis (SSIGN) Risk Score, and the University of California Los Angeles Integrated Staging System (UISS), are considered to be strong prognostic indicators [4–7], these do not yet lead to the desired accuracy in predicting patient outcome. For example, the *c*-index for SSIGN to predict disease recurrence is 0.82, indicating that there is room for improvement. [8]. In addition, there have been several changes in the landscape of RCC management since the inception of such models. Therefore, there is a need to identify and develop additional molecular prognostic markers that can enhance the predictive performance of current prognostic models [5, 9–13].

In the search for prognostic markers, DNA methylation in particular has received recognition as epigenetic alterations are more frequently found in RCC compared to genetic alterations [14]. Despite this attention, DNA methylation biomarkers have not yet bridged the gap between laboratory and clinic [10], possibly due to lack of uniformity among the different studies [10, 15]. Particularly, observed disparities in genomic location of the methylation assay are suggested to have implications for the development of DNA methylation markers and their translation into clinic [16, 17].

To review the current evidence on methylation markers in RCC, we previously performed a systematic review and identified *GREM1, RASSF1A, GATA5, LAD1, NEFH, SCUBE3, PCDH8, SFRP1,* and *BNC1* as the most promising prognostic methylation markers for RCC at the moment [15]. We also previously identified a DNA methylation marker panel consisting of *GREM1, LAD1, NEURL*, and *NEFH* that predicts disease outcome for ccRCC patients [18].

Despite the fact that these markers, and approximately 70 other potential prognostic methylation markers for RCC, have been published over the last decades [15], a direct comparison of the performance of all these markers is lacking and none of these biomarkers is currently used in clinical practice. In this study, we performed a head-to-head comparison of the most promising methylation markers identified in literature using new technical assays designed to cover the most clinically relevant methylated genomic regions [16]. Prognostic value of the markers was assessed using multivariable prediction modeling in a series of ccRCC cases derived from the prospective Netherlands Cohort Study (NLCS). The performance of the final model was further validated in non-metastatic ccRCC cases collected from the archives of the Department of Pathology, Universtity Hospitals Leuven and in a series of ccRCC cases from The Cancer Genome Atlas (TCGA).

## Materials and methods

### Methylation specific PCR assay development

In our systematic review on prognostic methylation markers in RCC [15], we did not only observe extensive heterogeneity regarding the methylation analysis techniques used across the different studies, but also regarding the exact genomic location that was used to asses a particular gene. It is known that the clinical value of DNA methylation may differ according to the exact genomic location that is analyzed, even within the gene promoter region, which complicates head-to-head comparison of DNA methylation markers’ performance and hampers subsequent clinical translation [16, 17, 19]. We therefore applied the approach that we recently developed using publicly available DNA methylation data from The Cancer Genome Atlas (TCGA) [16] to determine the genomic location of methylation that is most likely to be clinically relevant and thus represents the optimal location for methylation assay development. Methylation data from ccRCC samples and normal kidney samples from TCGA were plotted to gain insight in the promoter CpG-island methylation pattern of each gene. Primers for methylation-specific PCR (MSP) were designed in regions in which there was statistically significant differential methylation between tumor and normal samples, and normal samples had a methylation level (i.e. the *β*-value) lower than 0.2 (see Additional file 1: Fig. S1 for examples).

Our previously designed MSP assays for *GREM1* (three different regions [19]), *LAD1, NEFH* and *NEURL* were also re-evaluated to see whether we could further improve these assays. All our previously designed MSP assays were located at genomic regions that complied to the above-described criteria (data not shown). However, for *LAD1, NEFH*, and *NEURL* an additional, potentially clinically relevant promoter region was identified for which new assays were designed. Also, for *PCDH8, BNC1*, and *SCUBE3* two clinically relevant regions were identified and two different assays were developed. However, the primer sets for *BNC1* region i and *SCUBE3* region ii failed optimization and were not used in further analyses.

### Study population

Formalin-fixed paraffin-embedded (FFPE) primary ccRCC tissue samples were obtained from the NLCS, a prospective, population-based cohort study. This study was initiated in 1986 and included 120,852 men and women in the ages of 55–69 years at baseline [20]. After 20.3 years of follow-up, 608 incident RCC cases were identified and tumor tissues were collected from 51 pathology laboratories throughout the Netherlands. Tissue collection was conducted in two phases: initially only tumor samples of cases identified in the first 11.3 years of follow-up were included, but recently the series was expanded to 20.3 years of follow-up. Tumor tissue was available for 453 of the identified RCC cases. Hematoxylin and eosin (HE) stained slides of tumor tissues were revised by two experienced genitourinary pathologists to confirm tumor histology and Fuhrman grade. Information on patient and tumor characteristics (i.e. sex, age at diagnosis, TNM stage, and tumor size) was derived from the pathology reports and the cancer registry. TNM stage was classified according to the 1987 version of the TNM classification [21], as recoding to more recent TNM classifications was not possible due to a lack of needed information. Difference in tumor size between the 1987 version and more recent TNM classifications was addressed by including tumor size as a covariate in the statistical models. Follow-up was accomplished by record linkage to the causes of death registry from Statistics Netherlands and the municipal population registries. Patients included for this study had not received adjuvant therapy and further details of the tissue collection and follow-up have been described in detail elsewhere [22].

For this study, only patients with histologically confirmed ccRCC, with non-metastatic disease at diagnosis were included (*n* = 336). This led to the inclusion of some patients with TNM stage IV disease (*n* = 9) but with M0 regarding distant metastasis. The clinicopathological characteristics of the included patients are summarized in Additional file 1: Table S1 and S2.

For the validation of our proposed prognostic model, we used FFPE ccRCC tissue samples (*n* = 64) from the archives of the Department of Pathology, University Hospitals Leuven. These tumor samples were collected prospectively from patients with sporadic ccRCC, treated with radical or partial nephrectomy without any neo-adjuvant therapy, and are further referred to as the hospital-based series. All HE slides were revised by an experienced genitourinary pathologist. The clinicopathological characteristics of the included patients are summarized in Additional file 1: Table S3.

### The Cancer Genome Atlas data analysis

A patient series with the same clinicopathological characteristics (i.e. ccRCC subtype, non-metastatic disease) with a median follow up of 39 months was derived from TCGA, and was used as an additional validation cohort (Additional file 1: Table S4). DNA promoter methylation was measured using the Illumina Human Methylation 450 K platform. For each of the five genes included in the final model, the probes located closest to, or within our primer region, were identified (Additional file 1: Table S5). For genes for which more than one probe was identified, the methylation level was determined by calculating the mean *β*-value of these probes. We defined methylated samples as those with a (mean) *β*-value of > 0.2.

### DNA isolation and sodium bisulfite conversion

Genomic DNA from five 20-μm slices from FFPE ccRCC tissue from the population-based series, collected during the first 11.3 years of follow-up, was isolated as follows: paraffin was removed with xylene and genomic DNA was extracted by salt-precipitation. Briefly, 450 μl of cell lysis solution (10 mM Tris/HCl (pH 7.4), 400 mM NaCl, 2 mM EDTA), 25 μl of 10% SDS and 50 μl of proteinase K solution (20 mg/ml) were added to the tissue samples and incubated over-night at 55 °C. Proteins were precipitated using 175 μl of saturated NaCl, followed by centrifugation (2 min, 13.200 rpm). DNA was precipitated by the addition of 0.6 volumes of iso-propanol, dissolved in TE (pH 7.4) and stored at − 20 °C [23]. The isolated DNA was further cleaned up using QIAamp DNA Mini Kit (Qiagen, Venlo, the Netherlands) according to the manufacturer’s instructions. Genomic DNA from four 20 μm slices samples collected in the second phase of follow-up was isolated after macro-dissection using the QIAamp DNA Mini Kit (Qiagen, Venlo, the Netherlands) according to the manufacturer’s instructions. To isolate genomic DNA from the ccRCC hospital-based series FFPE samples, we first cut ten 10-μm slices, incubated the slides overnight at 37 °C and deparaffinized the sections by incubation for 5 min on Xylene twice. Afterwards, the slides were incubated twice for 3 min in 100% Ethanol, followed by an incubation for 1 min in 96% Ethanol and for 1 min in 70% Ethanol. Genomic DNA was thereafter isolated using the QIAamp DNA FFPE Tissue kit according to manufacturer’s protocol. The quality and concentration of the extracted DNA was estimated using NanoDrop quantification (NanoDrop ND-2000 m Spectrophotometer). To further evaluate the DNA integrity of our samples, we performed DNA integrity test on a subset of samples (Additional file 1: Table S12). In the population-based series, sodium bisulfite modification of 500 ng genomic DNA was performed using the EZ DNA Methylation Kit (Zymo Research, California, USA) according to the manufacturer’s instructions. In the hospital-based series, sodium bisulfite conversion of 500 ng genomic DNA was performed using EpiTect Bisulfite kit (Qiagen, Venlo, the Netherlands) according to manufacturer’s instructions.

### Promoter CpG island methylation analysis

Promoter CpG-island methylation was determined by nested MSP [24].To facilitate MSP analysis on DNA derived from FFPE material, DNA was first amplified with flanking PCR primers. Details on MSP PCR protocol and conditions are described in Additional file 1: Table S6, S7, S8 and S9. All primer sequences are provided in Additional file 1: Table S10. All PCR reactions were performed with controls for unmethylated alleles (CpGenomeTM Human Non-Methylated DNA Standard, MerckMillipore, USA or EpiTect control DNA unmethylated, Qiagen, Venlo, The Netherlands), methylated alleles (CpGenomeTM Human Methylated DNA Standard, MerckMillipore, USA or an in-house prepared in vitro methylated DNA control), and a no-template control without DNA. Ten µl of each MSP reaction was directly loaded onto 3% agarose gels containing Midori Green Advance DNA Stain (Nippon Genetics Europe, Dueren, Germany) and visualized under UV illumination. Reproducibility of MSP analysis was assessed by repeating 10% of the cases. Representative agarose gel examples of MSP experiments are shown in Additional file 1: Fig. S2, S3, S4 and S5.

### Statistical analysis

Cause-specific survival (CSS) was defined as time from cancer diagnosis until RCC-related death or end of follow-up. Analyses were restricted to 10 years after diagnosis as deaths related to RCC are not likely after that period.

Univariable survival analyses for all potential methylation markers separately were performed using Kaplan–Meier and log rank tests. Hazard ratios (HR) and corresponding 95% confidence intervals (CI) were determined with Cox proportional hazards models adjusted for potential confounders. Factors were considered possible confounders if they were known prognostic factors for RCC and included age at diagnosis (continuous), sex, and tumor size (mm). TNM staging was analyzed as a categorical factor stratifying into stage I, II, III and IV. Moreover, Fuhrman grading was also analyzed as a categorical factor distinguishing grade 1, 2, 3 and 4. To build a multivariable prediction model containing multiple methylation markers, we performed a backward stepwise elimination procedure using the likelihood-ratio test and a liberal *α* of 0.1 to prevent the exclusion of potentially important predictors from the model [25]. This was done to identify the most predictive combination of risk factors for survival of patients with non-metastatic ccRCC. Therefore, all known RCC prognostic factors (age at diagnosis, sex, TNM stage, tumor grade, and tumor size) were included in the model regardless of statistical significance. Continuous variables were included as such. We used restricted cubic splines to assess evidence of a non-linear association with the log-hazard of an event. The final model was compared with a model containing the known RCC prognostic factors (age at diagnosis, sex, TNM stage, Fuhrman grade, and tumor size) only. Model performance was assessed using Harrell’s c-statistic and Akaike’s Information Criteria (AIC). The preferred model was the one with the lowest AIC and the highest c-statistic (c-statistic was leading if the highest c-statistic did not have the lowest AIC). The initial prediction model was internally validated using bootstrapping (number of bootstraps was 1000) [25]. Results from this validation step were used to penalize the regression coefficients to prevent too optimistic predictions and to estimate an optimism-corrected c-statistic.

To visualize model performance in our dataset, risk scores for individual patients were computed based on the final model and were used to split the data into 3 subgroups based on tertiles. Kaplan–Meier curves were created for all subgroups. All analyses were performed using the statistical software STATA 14.1 and R 3.4.1.

## Results

### Patient characteristics and epigenetic characterization of ccRCCs

Additional file 1: Table S1 and S2 show the baseline characteristics of the population-based series. The majority of the patients was male (58.6%), and the mean age at diagnosis was 71.2 years. The median ccRCC-specific survival in the total population was 8.1 years. During 10 years follow-up, 122 patients died of a renal cancer-related cause. The frequency of promoter methylation of the studied genes ranged from 18% (*SFRP1*) to 84% (*RASSF1A*) (Additional file 1: Fig. S6 and Table S11). For *PCDH8, LAD1, NEFH*, and *NEURL* two, and in the case of *GREM1* three, different locations within the promoter region were analyzed. The methylation frequencies of *NEURL, LAD1*, and *GREM1* slightly differed according to the region analyzed (*NEURL-i* 39% vs. *NEURL-ii* 35%, *LAD1-i* 28% vs. *LAD1-ii* 37%, and *GREM1-i* 29% vs. *GREM1-ii* 42% vs. *GREM1-iii* 40%). For *PCDH8* no difference in methylation frequency was observed, while for *NEFH* the methylation frequency greatly varied between the two regions analyzed (*NEFH-i* 28% vs. *NEFH-ii* 69%) (Additional file 1: Fig. S6 and Table S11).

### Clinical value of DNA methylation is location dependent

Next, we used Kaplan–Meier analysis to examine the prognostic value of the 11 candidate DNA methylation markers (including the different genomic locations, in total 17 assays) in the population-based series of non-metastatic ccRCC cases (Additional file 1: Fig. S7 and Fig. S8). The methylation status of *GREM1* (region i), *LAD1* (region i and ii), *NEFH* (region i), *NEURL* (region ii), *SFRP1*, and *GATA5* was significantly associated with poorer ccRCC-specific survival (log-rank test, *P* ≤ 0.0001–0.0117, Additional file 1: Fig. S7). Notably, the association between methylation of *GREM1, NEFH, NEURL* and patient outcome was dependent on the region that is methylated. For *GREM1*, only methylation of the region located most upstream of the TSS (*GREM1-i*) was associated with patient outcome (log-rank test, *P* = 0.0022), while for the other two regions no significant association with survival was found (log-rank test, *GREM1-ii* *P* = 0.5662 and *GREM1-iii* *P* = 0.2102). Also, for *NEFH* and *NEURL*, methylation of only one of the two studied regions was associated with ccRCC-specific survival (log-rank test, *NEFH-i* *P* = 0.0067 versus *NEFH-ii* *P* = 0.9680 and *NEURL-i* *P* = 0.1533 versus *NEURL-ii* *P* = 0.0010).

Multivariate Cox proportional hazards analyses showed that advanced disease stage and Fuhrman grade were independent, statistically significant predictors of poor survival (Additional file 1: Table S11). Subsequently, the prognostic value of the individual methylation markers was assessed in a multivariate model together with known prognostic variables (i.e. TNM stage, Fuhrman grade, and tumor size) including age at diagnosis and gender. In multivariate analyses, methylation of *GREM1-i, LAD1-i, LAD1-ii, NEFH-i, NEURL-ii, SFRP1,* and *GATA5* remained statistically significant predictors of poor survival (HR_*GREM1-i*_ 1.86 (95% CI 1.16–2.97), HR_*LAD1-i*_ 2.26 (95% CI 1.47–3.48), HR_*LAD1-ii*_ 1.71 (95% CI 1.12–2.61), HR_NEFH*-i*_ 1.74 (95% CI 1.11–2.74), HR_*NEURL-ii*_ 1.94 (95% CI 1.21–3.13), HR_*SFRP1*_ 1.89 (95% CI 1.16–3.08), and HR_*GATA5*_ 1.67 (95% CI 1.08–2.60) (Additional file 1: Table S11).

### A prognostic risk model for patients with non-metastatic ccRCC

**Fig. 1:**
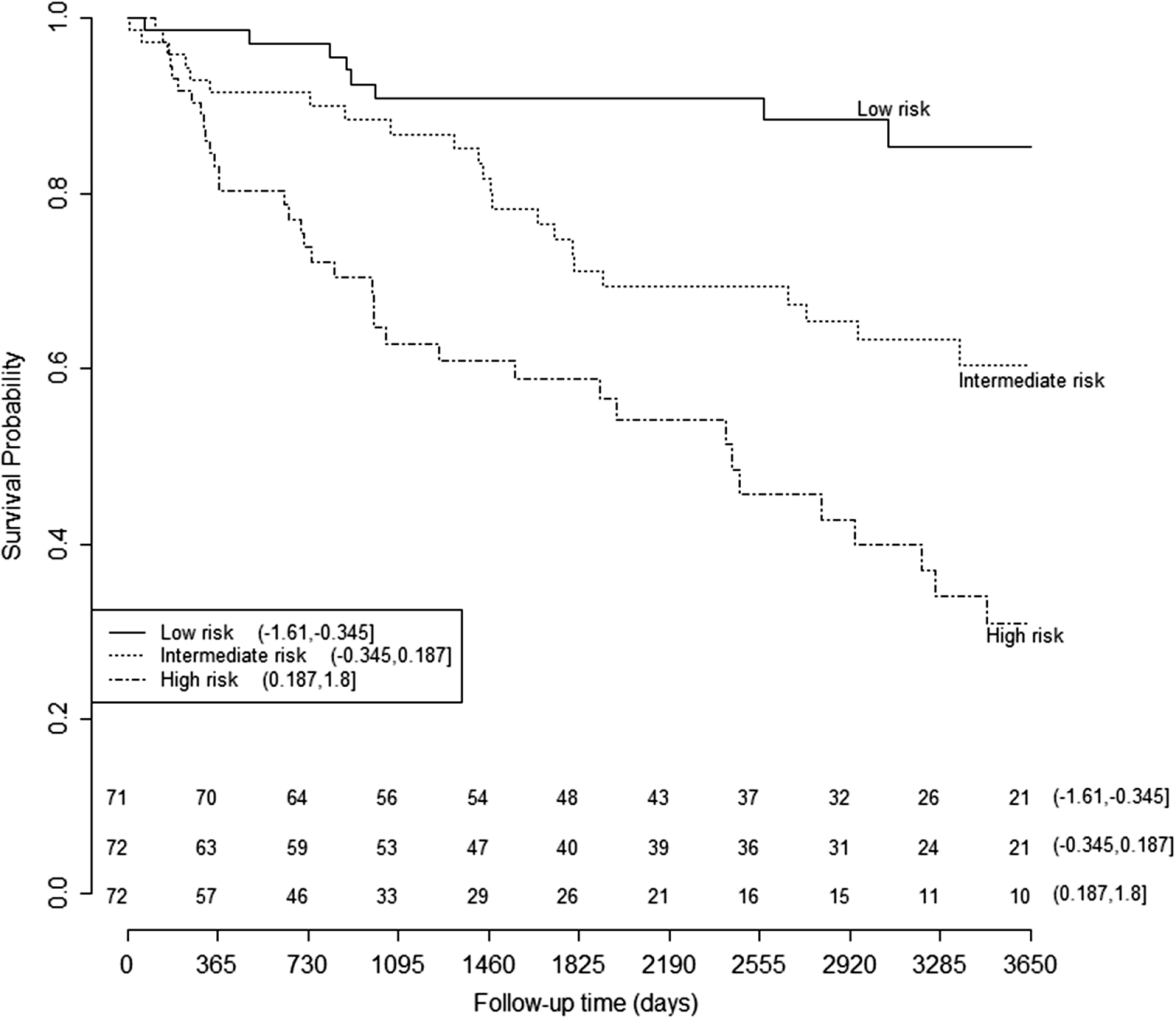
Risk score calculated by final model and survival curves in the population-based ccRCC series. Kaplan–Meier curves for overall cause-specific survival based on risk score. Patients were divided into low-, intermediate-, and high-risk groups based on tertiles

We next sought to determine whether these prognostic methylation markers could add prognostic value to the clinical variables that are currently used to predict prognosis (i.e. TNM stage, Fuhrman grade, and tumor size). The final prognostic risk model consisted of the standard prognostic variables and five methylation markers (i.e. *NEFH-ii, GREM1-ii, GATA5, NEURL-*ii and *LAD1-ii*). TNM stage, Fuhrman grade, and methylation of *LAD1-ii* were the most significant predictors of survival. All coefficients and HRs for the final model are presented in Table 1. The c-statistic for the final model was 0.71 (0.65 after correction for optimism), with an AIC of 674. Comparison with the clinical model containing the standard prognostic variables alone (c-statistic 0.65, AIC 681) showed that model fit and performance were better for our final prognostic model including the five methylation markers (Table 2). Kaplan–Meier curves were generated for the final model, showing a low-, intermediate- and high-risk group that could be identified with the prognostic model including the five methylation markers (Fig. 1).

**Table 1:** Hazard ratios for the final model in non-metastatic ccRCC (population-based series)

Marker	Values	Coef	SE	HR (95% CI)	*P*-value
Age at diagnosis	Continuous (yrs)	0.021	0.023	1.02 (0.98–1.07)	0.36
Gender	Male			1 (ref)	
	Female	0.109	0.272	1.11 (0.65–1.90)	0.69
TNM stage^a^	I			1 (ref)	
	II	0.569	0.638	1.77 (0.51–6.17)	0.37
	III	1.207	0.685	3.34 (0.87–12.79)	0.08
	IV	1.884	0.915	6.58 (1.09–39.57)	0.04
Fuhrman grade	G1			1 (ref)	
	G2	-0.112	0.426	0.89 (0.39–2.06)	0.79
	G3	-0.047	0.437	0.95 (0.41–2.25)	0.92
	G4	0.955	0.462	2.60 (1.05–6.43)	0.04
Tumor size	Continuous (mm)	-0.003	0.005	1.00 (0.99–1.01)	0.59
*NEFH-ii*	U			1 (ref)	
	M	-0.002	0.298	1.00 (0.56–1.79)	0.99
*NEURL-ii*	U			1 (ref)	
	M	0.475	0.280	1.61 (0.93–2.78)	0.09
*LAD1-ii*	U			1 (ref)	
	M	0.738	0.258	2.07 (1.25–3.43)	0.01
*GREM1-ii*	U			1 (ref)	
	M	-0.456	0.284	0.64 (0.36–1.11)	0.11
*GATA5*	U			1 (ref)	
	M	0.351	0.283	1.43 (0.82–2.49)	0.20

Coef, coefficient; HR, hazard ratio; M, methylated; ref, reference; SE, standard error; TNM, tumor-node-metastasis; U, unmethylated; yrs, years; 95% CI, 95% confidence interval

^a^TNM stage as defined in 1987

**Table 2:** Comparison of model performance and fit

Models	Population-based series				Hospital-based series				TCGA series			
	*n*	Df	AIC	C-statistic	*n*	Df	AIC	C-statistic	*n*	Df	AIC	C-statistic
Clinical model^a^	219	9	681	0.65	42	5	63	0.86	227	7	470	0.75
Prognostic model^b^	219^c^	14	674	0.71	42	10	55	0.95	227	12	475	0.76

^a–c^Performance of both the clinical Cox proportional hazards model a (including age at diagnosis, sex, Fuhrman grade, tumor size and TNM stage) and the prognostic biomarker Cox proportional hazards model b (containing age at diagnosis, gender, Fuhrman grade, tumor size, TNM stage, methylation of *NEFH, GREM1, GATA5, LAD1,* and *NEURL*). Numbers in the table refer to the number of cases included in the analysis (*n*), degrees of freedom (Df), Akaike Information Criterion (AIC) and the Harrel’s C-statistic (C-statistic). c Lower number of patients due to missing data on methylation status of the included genes

### Validation of the prognostic risk model in the hospital-based series

Additional file 1: Table S3 shows the baseline characteristics of the hospital-based series. Compared to the population-based series, patients in the hospital-based series had a significantly lower mean age at diagnosis (59.3 ± 12.1 versus 71.2 ± 6.3 years). In both series, the majority of patients were diagnosed with stage II disease (61.9% and 59.6%). Except for the methylation of *GREM1-ii*, methylation frequencies of the genes included in the final model were comparable between the hospital-based and the population-based series (Additional file 1: Table S1, S2 and S3). Notably, performance of our final prognostic model in the hospital-based series was significantly better than its performance in the population-based series, with a c-statistic of 0.95 versus 0.71. Comparably, the model including only standard prognostic variables seemed to have a better performance in the hospital-based series with a c-statistic of 0.86 versus a c-statistic of 0.65 in the population-based series (Table 2). However, comparison of the clinical model with the final prognostic model in the hospital-based series revealed that model fit and performance were better for the final model, indicating that the addition of methylation markers to the clinical model provides additional prognostic value (Table 2).

### Validation of the prognostic model in the TCGA series

Additional file 1: Table S4 shows the baseline characteristics of the TCGA series. Compared to the population-based series, patients in the TCGA series also had a lower mean age at diagnosis (61.7 ± 12.3 versus 71.2 ± 6.3), and considerably more patients were diagnosed with stage I disease (56.0% versus 5.7%), and hence fewer with stage II. Methylation frequencies of the five genes included in the model differed between the population-based and the TCGA series, with generally lower methylation frequencies in the TCGA series, except for methylation of *NEURL-ii* which was found to be methylated in 95% of the TCGA cases compared to 35% in the population-based series (Additional file 1: Table S1, S2 and S4). In this respect, it has to be pointed out that methylation in TCGA is measured using a quantitative assay (i.e. the Illumina Human Methylation 450 K platform) and that for some genes the available probes were not exactly located within the MSP assay (see Additional file 1: Table S5).

Performance of our prognostic model with the methylation markers in the TCGA series was somewhat better than its performance in the population-based series, with a c-statistic of 0.76 versus 0.71. However, the model including only standard prognostic variables without the methylation markers showed a comparable performance to the prognostic model with (Table 2). Hence, addition of the methylation markers to the model provided limited incremental prognostic value in the TCGA series.

### Protein and mRNA expression data

No protein expression data was available for any of our markers in TCGA nor in the Central Proteomic Tumor Analysis Consortium. However, mRNA-seq data from TCGA revealed statistically significant differences between normal and tumor tissue (Mann–Whitney test, *P* < 0.05), as well as between normal and the various tumor stages (Kruskal–Wallis rank sum test, *P* < 0.05) for all the markers described (data not shown).

## Discussion

In the present study, we performed a head-to-head comparison of reported potential prognostic methylation markers for ccRCC and developed a prognostic risk model for non-metastatic ccRCC patients that includes standard clinicopathological features plus five methylation markers. Our model including the methylation markers might have additional prognostic value as compared to the model including only clinicopathological factors.

Up until now, a direct comparison of the performance of suggested prognostic methylation biomarkers from previous studies has not been feasible, due to large differences among studies, especially in the exact genomic location of DNA-methylation assays [26]. In order to facilitate a direct comparison for this study, we applied our in-house developed strategy to design methylation assays at the clinically most relevant genomic location for all studied biomarkers [16].

A total of 6 (i.e. *GREM1* (region i), *LAD1* (region i and ii), *NEFH* (region i), *NEURL* (region ii), *SFRP1*, and *GATA5*) out of the 11 published, promising methylation markers were validated as predictors of CSS in a population-based series of ccRCC patients independent of standard prognostic variables. Using multivariable prediction modeling we developed a prognostic risk model including the standard prognostic variables and five methylation markers (i.e. *NEFH-ii, GREM1-ii, GATA5, NEURL-ii* and *LAD1-ii*) that appears to have independent prognostic value in addition to the standard clinical and pathological variables.

Our model showed similar results in a second, hospital-based series with a better prognostic performance of the prognostic model over the clinical model. However, in TCGA, performance of the model including standard prognostic variables was comparable to that of the model including the methylation markers. There are however extensive differences between the three study populations. Firstly, the population-based series has been prospectively collected from a representative base population, including up to 80% of all incident ccRCC cases. In contrast, the hospital-based series and the TCGA series represent a selected study population and, in the case of the TCGA series, with less detailed clinical and follow up data. Moreover, considerably more patients were diagnosed with stage I disease in the TCGA series compared to the population-based and hospital-based series. This could however be due to differences in the TNM version used. In the population-based series patients were staged according to the TNM classification from 1987. Conversion to the latest TNM classification was not possible due to lack of information in the cancer registry files and the pathological reports. Because the main difference between the TNM classifications is the cut-off values for tumor size, we added tumor size as a separate variable in our analyses. In addition to that, methylation in the TCGA is measured using the Illumina Human Methylation 450 K platform as a quantitative trait which forced us to set a cut-off value for methylation above a *β*-value of 0.2. Although this is a commonly used value, altering this threshold may influence the methylation status of the samples and eventual conclusions. This could explain the disparities in methylation frequencies between the population-based, hospital-based and TCGA series.

Interestingly, for *LAD1, NEFH*, and *NEURL*, only one of the designed MSP assays was retained in the final model. This suggests that only methylation at that specific region has independent prognostic value. Traditionally, research in the DNA methylation marker field has focused on hypermethylation of the promoter region of genes [27] and undervalued the fact that not all CpGs in the promoter region are functionally uniform [16, 28]. The importance of the genomic location of DNA methylation markers and its biological and clinical relevance has previously been postulated [17, 19]. Here, we therefore utilized publicly available DNA methylation data from TCGA to select the optimal location for DNA methylation-based biomarker development [16] for each of the genes. Results suggest that for *GREM1, NEFH,* and *NEURL* the association between methylation and patient outcome was indeed dependent on the chosen assay location, supporting the fact that the location of DNA methylation influences the clinical relevance of the marker. These observations further highlight that the location of the CpG dinucleotides to be analyzed and with this namely MSP primer location and design can influence the observed methylation frequency and clinical relevance. Moreover, this highlights that developing an optimal methylation assay should not be solely dependent on selecting the optimal methylation location but also on combining that with clinical outcome factors.

We further explored the biological relevance of these markers and found that *GREM1* is a member of the cystine knot superfamily and a bone morphogenetic protein (BMP) antagonist [29, 30] and that it plays a crucial role in embryogenesis, tissue differentiation and organ development through the regulation of the BMPs [31]. Several studies revealed its involvement in renal inflammation [32, 33] and renal fibrosis [34] through the epithelia mesenchymal transition (EMT) process, which in turn is critical for tumor metastasis and cancer progression [35]. Additionally, *GREM1* stimulates angiogenesis and neovascularization by binding directly to vascular endothelial growth factor (VEGF) receptor 2 (VEGFR2) [36].

*GATA 5* is a zinc-finger transcription factor and member of the GATA family proteins 1–6 and is known to be involved in cellular differentiation [37]. It has been reported that hypermethylation of *GATA5* is associated with metastasis and progression‐free survival of RCC patients [38]. Besides that, several studies have shown that reduced *GATA 5* mRNA levels were associated with a poor clinical outcome, indicating a possible role of GATA 5 for the development of aggressive ccRCC phenotypes [37].

*LAD 1* is a relatively uncharacterized protein and is proposed to contribute to the stability of the association of the epithelial layers with the underlying mesenchyme. Therefore, it is believed that *LAD 1* is involved in cell adhesion, cytoskeleton organization and invasion [14, 39, 40].

*NEFH* gene encodes the heavy neurofilament protein [41] and is suggested to play a role in the cell motility [14, 42]. Moreover, in tumor cells, loss of *NEFH* by promoter methylation leads to increased aerobic glycolysis and mitochondrial dysfunction [43] and could, therefore, contribute to the metabolic shift that is observed in aggressive ccRCC [44].

The *NEURL* on the other hand acts as a tumor suppressor [45] and is involved in the Notch signaling pathway [18, 46] which is critical for determination of cell fates within a wide variety of tissues by regulation of growth, differentiation, and apoptosis. Furthermore, several studies revealed that the Notch signaling pathway has an important role in the development of the mammalian kidney whereby several key members of the Notch cascade are expressed during nephrogenesis [47].

Although our model including the five methylation markers showed some incremental prognostic value compared with standard clinicopathological variables alone, we were not able to evaluate whether the identified prognostic methylation markers were also able to further improve other established prognostic models such as the UISS and the SSIGN score [5], due to lack of information on tumor necrosis and patients’ performance status in our patient series. This would however be important as these prognostic models are now considered efficacious tools in tailoring RCC management and making decisions for selection for adjuvant therapy [48]. Moreover, we have developed our prognostic model using stepwise backward elimination which, despite being the most popular variable selection method, has its shortcomings [49]. For example, backward elimination can lead to bias in parameter estimation as once a variable is eliminated from the model it is not re-entered again, whilst a dropped variable may become significant later in the final model [49].

Furthermore, we were not able to assess the effect of intratumor heterogeneity (ITH) on the performance of our model, as no multi-region sampling was performed at the time of tumor tissue collection. ITH can impede biomarker development due to sampling bias [50, 51] and can affect the reproducibility of biomarkers when relying solely on a single area of the tumor [52]. However, in contrast to the extensive genetic ITH observed in RCC, epigenetic intratumor heterogeneity (eITH), in particular DNA methylation, seems to be less prominent [53]. It is warranted that multiregion sampling is performed in further validation studies of our proposed prognostic model, as this will contribute to elucidating a possible impact of ITH on the performance of our prognostic model.

## Conclusion

This work presents a head-to-head comparison of prognostic methylation markers for RCC using a novel approach to determine the optimal location for DNA methylation-based biomarker development. We demonstrated that using epigenomic TCGA data to guide methylation assay design is a suitable approach to identify clinically relevant regions of methylation. Using this concept, we were able to develop a prognostic risk model, including DNA methylation markers, for ccRCC that is suggested to have additional value in patients with localized ccRCC compared with the typical clinicopathological risk factors. This indicates that evaluating well defined and validated molecular markers for incremental value to existing models is worth the effort. However, further validation in large prospective series with extended patient data that comply with the requirements of the current prognostic models in practice, such as the SSIGN score and UISS, is crucial to establish its actual clinical value.

### Supplementary Information

**Additional file 1.** Supplementary Tables and Figures.

## Data Availability

All data generated or analyzed during this study are included in this published article [and its supplementary information files].
