# Supplementary material for: Development of a prognostic risk model for clear cell renal cell carcinoma by systematic evaluation of DNA methylation markers
Source: Clin Epigenetics. 2021 May 4;13:103. doi: 10.1186/s13148-021-01084-8 (PMC8094610; doi:10.1186/s13148-021-01084-8)
Supplement: Supplementary file 1 — Additional file 1. Supplementary Tables and Figures. [file 13148_2021_1084_MOESM1_ESM.docx]

SUPPLEMENTARY TABLES

| **SUPPL TABLE S1 \|** Characteristics of clear cell renal cell cancer patients overall and by promoter methylation of *GREM1-i, LAD1-i, LAD1-ii, NEFH-i, NEURL-ii, SFRP1,* and *GATA5* in the population-based series | | | | | | | | |
| --- | --- | --- | --- | --- | --- | --- | --- | --- |
|  | **All ccRCC**  **n=336** | **Methylated *GREM1-i***  **n=83 (29%)** | **Methylated *LAD1-i***  **n=87 (28%)** | **Methylated *LAD1-ii***  **n=111 (37%)** | **Methylated**  ***NEFH-i***  **n=81 (28%)** | **Methylated *NEURL-ii***  **n=101 (35%)** | **Methylated**  ***SFRP1***  **n=57 (18%)** | **Methylated *GATA5***  **n=96 (33%)** |
| Gender – no. (%) |  |  |  |  |  |  |  |  |
| Male | 197 (58.6) | 50 (60.2) | 57 (65.5) | 70 (63.1) | 54 (66.7) | 54 (53.5) | 35 (61.4) | 57 (59.4) |
| Female | 139 (41.4) | 33 (39.8) | 30 (34.5) | 41 (36.9) | 27 (33.3) | 47 (46.5) | 22 (38.6) | 39 (40.6) |
| *P*-value^a^ (U versus M) |  | 0.584 | 0.222 | 0.231 | 0.071 | 0.253 | 0.689 | 0.998 |
| Age - mean (SD) | 71.2 (6.3) | 71.2 (6.0) | 71.0 (6.4) | 70.7 (6.0) | 71.3 (6.6) | 72.2 (6.4) | 71.5 (7.1) | 72.2 (6.5) |
| *P*-value (U versus M) |  | 0.887 | 0.598 | 0.131 | 0.940 | 0.194 | 0.816 | 0.100 |
| Tumor size in mm – mean (SD) | 64.9 (31.3) | 69.2 (38.5) | 68.9 (35.6) | 69.4 (32.6) | 68.7 (28.6) | 69.3 (32.6) | 76.8 (31.2) | 74.1 (36.1) |
| *P* (U versus M) |  | 0.442 | 0.296 | 0.142 | 0.389 | 0.119 | 0.003 | 0.003 |
| Fuhrman grade – no. (%) |  |  |  |  |  |  |  |  |
| G1 | 45 (13.4) | 12 (14.5) | 10 (11.5) | 10 (9.0) | 9 (11.1) | 5 (4.9) | 4 (7.0) | 9 (9.4) |
| G2 | 139 (41.4) | 28 (33.7) | 35 (40.2) | 39 (35.1) | 28 (34.6) | 41 (40.6) | 16 (28.1) | 37 (38.5) |
| G3 | 107 (31.9) | 27 (32.5) | 28 (32.2) | 45 (40.5) | 35 (43.2) | 34 (33.7) | 21 (36.8) | 34 (35.4) |
| G4 | 45 (13.4) | 16 (19.3) | 14 (16.1) | 17 (15.3) | 9 (11.1) | 21 (20.8) | 16 (28.1) | 16 (16.7) |
| *P*-value (U versus M) |  | 0.116 | 0.646 | 0.004 | 0.134 | 0.001 | <0.0001 | 0.346 |
| T – no. (%) |  |  |  |  |  |  |  |  |
| 1 | 19 (5.7) | 6 (7.2) | 8 (9.2) | 8 (7.2) | 5 (6.2) | 7 (6.9) | 1 (1.8) | 4 (4.2) |
| 2 | 203 (60.4) | 43 (51.8) | 38 (43.7) | 55 (49.5) | 37 (45.7) | 56 (55.4) | 26 (45.6) | 57 (59.4) |
| 3 | 112 (33.3) | 33 (39.8) | 40 (46.0) | 46 (41.4) | 38 (46.9) | 37 (36.6) | 30 (52.6) | 34 (35.4) |
| 4 | 1 (0.3) | 0 (0.0) | 0 (0.0) | 1 (0.9) | 0 (0.0) | 1 (1.0) | 0 (0.0) | 1 (1.0) |
| Not available | 1 (0.3) | 1 (1.2) | 1 (1.1) | 1 (0.9) | 1 (1.2) | 0 (0.0) | 0 (0.0) | 0 (0.0) |
| *P*-value (U versus M) |  | 0.262 | 0.003 | 0.105 | 0.023 | 0.525 | 0.005 | 0.444 |
| N – no. (%) |  |  |  |  |  |  |  |  |
| 0 | 248 (73.8) | 64 (77.1) | 64 (73.6) | 81 (73.0) | 58 (71.6) | 73 (72.3) | 34 (59.6) | 69 (71.9) |
| 1 | 88 (26.2) | 19 (22.9) | 23 (26.4) | 30 (27.0) | 23 (28.4) | 28 (27.7) | 23 (40.4) | 27 (28.1) |
| *P*-value (U versus M) |  | 0.632 | 0.564 | 0.045 | 0.763 | 0.666 | 0.025 | 0.813 |
| TNM stage^b^ – no. (%) |  |  |  |  |  |  |  |  |
| I | 19 (5.7) | 6 (7.2) | 8 (9.2) | 8 (7.3) | 5 (6.2) | 7 (6.9) | 1 (1.8) | 4 (4.2) |
| II | 199 (59.6) | 42 (50.6) | 37 (42.5) | 52 (46.8) | 36 (44.4) | 54 (53.5) | 25 (43.9) | 56 (58.3) |
| III | 107 (32.0) | 32 (38.6) | 39 (44.8) | 46 (41.4) | 38 (47.0) | 38 (37.6) | 30 (52.6) | 33 (34.4) |
| IV | 9 (2.7) | 1 (1.2) | 1 (1.2) | 3 (2.7) | 1 (1.2) | 2 (2.0) | 1 (1.8) | 2 (2.1) |
| Not available | 2 (0.6) | 2 (2.4) | 2 (2.3) | 2 (1.8) | 1 (1.2) | 0 (0.0) | 0 (0.0) | 1 (1.0) |
| *P*-value (U versus M) |  | 0.273 | 0.001 | 0.058 | 0.007 | 0.569 | 0.002 | 0.853 |
| ^a^ *P*-value tested with two-sample *t* test (for continuous variables) or χ^2^ test (for categorical variables); ^b^ TNM stage as defined in 1987. U: unmethylated; M: methylated; SD: standard deviation | | | | | | | | |

| **SUPPL TABLE S2 \|** Characteristics of clear cell renal cell cancer patients by promoter methylation of *GREM1-ii, GREM1-iii, NEFH-ii, NEURL-i, PCDH8-i, PCDH8-i, PCDH8-ii, SCUBE3, BNC1, CDO1 and RASFF1A* in the population-based series | | | | | | | | | | |
| --- | --- | --- | --- | --- | --- | --- | --- | --- | --- | --- |
|  | **Methylated *GREM 1-ii***  **n=126 (42.3%)** | **Methylated *GREM 1-iii***  **n=125 (39.8%)** | **Methylated *NEFH-ii***  **n=190 (68.6%)** | **Methylated**  ***NEURL-i***  **n=128 (39.4)** | **Methylated *PCDH8-i***  **n=230 (79.0%)** | **Methylated** ***PCDH8-ii***  **n=243 (81.2%)** | ***Methylated SCUBE3***  ***n=234(80.1%)*** | ***Methylated BNC1***  ***n=158 (54.3%)*** | ***Methylated CDO1***  ***n=118 (38.6%)*** | ***Methylated***  ***RASFF1A***  ***n=251 (83.6%)*** |
| Gender – no. (%) |  |  |  |  |  |  |  |  |  |  |
| Male | 71 (56.3) | 75 (60) | 104 (54.7) | 78 (60.9) | 130 (56.5) | 136 (56) | 133(56.8) | 90 (62) | 84 (71.2) | 147 (58.6) |
| Female | 55 (34.4) | 50 (40) | 86 (45.3) | 50 (39.1) | 100 (43.5) | 107 (44) | 101(43.2) | 68 (43) | 34 (28.8) | 104 (41.4) |
| *P*-value^a^ (U versus M) | 0.359 | 0.040 | 0.874 | 0.646 | 0.202 | 0.167 | 0.806 | 0.675 | 0.000 | 0.476 |
| Age – mean (SD) | 72.3 (6.1) | 71.1 (6.7) | 72 (6.4) | 70.8 (6.1) | 71.6 (6.1) | 71.5 (6.3) | 71.7 (6.1) | 72.4 (6.05) | 71.5 (6.2) | 71.2 (6.4) |
| *P*-value (U versus M) |  | 0.664 | 0.180 | 0.336 | 0.390 | 0.753 | 0.055 | 0.016 | 0.601 | 0.716 |
| Tumor size in mm – mean (SD) | 65 (31.9) | 68.4 (32.7) | 65.5 (32.6) | 68.6 (31.1) | 65.9 (30.5) | 64.4 (29.5) | 64.9 (31.7) | 66.9 (32.2) | 68 (33.5) | 66.2 (32.2) |
| *P*-value (U versus M) | 0.931 | 0.143 | 0.726 | 0.154 | 0.798 | 0.096 | 0.368 | 0.310 | 0.314 | 0.295 |
| Fuhrman grade – no. (%) |  |  |  |  |  |  |  |  |  |  |
| G1 | 15 (11.9) | 19 (15.2) | 22 (11.6) | 15 (11.7) | 29 (12.6) | 31 (12.8) | 26 (11.1) | 21 (13.3) | 5 (4.2) | 35 (13.9) |
| G2 | 53 (42.1) | 39 (31.2) | 78 (41.0) | 53 (41.4) | 95 (41.3) | 104 (42.8) | 98 (41.9) | 62 (39.2) | 46 (39.1) | 107 (42.6) |
| G3 | 40 (31.7) | 45 (36.0) | 65 (34.2) | 43 (33.6) | 72 (31.3) | 77 (31.7) | 80 (34.2) | 52 (32.9) | 45 (38.1) | 78 (31.1) |
| G4 | 18 (14.3) | 22 (17.6) | 25 (13.2) | 17 (13.3) | 34 (14.8) | 31 (12.8) | 30 (12.8) | 23 (14.6) | 22 (18.6) | 31 (12.4) |
| Not Available | 0 (0.0) | 0 (0.0) | 0 (0.0) | 0 (0.0) | 0 (0.0) | 0 (0.0) | 0 (0.0) | 0 (0.0) | 0 (0.0) | 0 (0.0) |
| *P*-value (U versus M) | 0.736 | 0.010 | 0.689 | 0.852 | 0.380 | 0.866 | 0.049 | 0.856 | 0.000 | 0.831 |
| T – no. (%) |  |  |  |  |  |  |  |  |  |  |
| 1 | 8 (6.3) | 7 (5.6) | 13 (6.8) | 5 (3.9) | 12 (5.2) | 15 (6.1) | 15 (6.4) | 9 (5.7) | 8 (6.8) | 13 (5.2) |
| 2 | 70 (55.6) | 69 (55.2) | 113 (59.5) | 73 (57.0) | 139 (60.5) | 152 (62.6) | 143 (61.1) | 96 (60.8) | 65 (55.1) | 151 (60.2) |
| 3 | 47 (37.3) | 48 (38.4) | 62 (32.6) | 49 (38.3) | 77 (33.5) | 75 (30.9) | 75 (32.1) | 52 (32.9) | 44 (37.3) | 86 (34.3) |
| 4 | 0 (0.0) | 1 (0.8) | 1 (0.5) | 0 (0.0) | 1 (0.4) | 1 (0.4) | 1 (0.4) | 1 (0.6) | 0 (0.0) | 1 (0.4) |
| Not available | 1 (0.8) | 0 (0.0) | 1 (0.5) | 1 (0.8) | 1 (0.4) | 0 (0.0) | 0 (0.0) | 0 (0.0) | 1 (0.8) | 0 (0.0) |
| *P*-value (U versus M) | 0.596 | 0218 | 0.169 | 0.525 | 0.880 | 0.261 | 0.319 | 0.737 | 0.230 | 0.789 |
| N – no. (%) |  |  |  |  |  |  |  |  |  |  |
| 0 | 96 (76.2) | 91 (72.8) | 143 (75.3) | 91 (71.1) | 172 (74.8) | 182 (74.9) | 182 (77.8) | 114 (72.2) | 87 (73.7) | 187 (74.5) |
| 1 | 30 (23.8) | 34 (27.2) | 47 (24.7) | 37 (28.9) | 58 (25.2) | 61 (25.1) | 52 (22.2) | 44 (27.8) | 31 (26.3) | 64 (25.5) |
| *P*-value (U versus M) | 0.081 | 0.937 | 0.667 | 0.609 | 0.703 | 0.439 | 0.092 | 0.430 | 0.966 | 0.173 |
| TNM stage^b^ – no. (%) |  |  |  |  |  |  |  |  |  |  |
| I | 8(5.6) | 7(5.6) | 13(6.8) | 5(3.9) | 12(5.2) | 15(6.2) | 15 (6.4) | 9 (5.8) | 8 (6.8) | 13 (5.2) |
| II | 70(55.6) | 68(54.4) | 112(59.0) | 72(56.2) | 136(59.2) | 150(61.7) | 140 (59.8) | 94 (59.5) | 63 (53.4) | 147 (58.5) |
| III | 47(37.3) | 46(36.8) | 60(31.6) | 47(36.7) | 74(32.2) | 73(30.0) | 74 (31.6) | 50 (31.6) | 43 (36.4) | 85 (33.9) |
| IV | 0(0.0) | 3(2.4) | 4(2.1) | 3(2.3) | 7(3.0) | 5(2.1) | 3 (1.3) | 4 (2.5) | 2 (1.7) | 4 (1.6) |
| Not available | 1(0.7) | 1(0.8) | 1(0.5) | 1(0.9) | 1(0.4) | 0(0.0) | 2 (0.9) | 1 (0.6) | 2 (1.7) | 2 (0.8) |
| *P*-value (U versus M) | 0.273 | 0.300 | 0.276 | 0.288 | 0.772 | 0.246 | 0.247 | 0.896 | 0.251 | 0.740 |
| ^a^ *P*-value tested with two-sample *t* test (for continuous variables) or χ^2^ test (for categorical variables); ^b^ TNM stage as defined in 1987. U: unmethylated; M: methylated; SD: standard deviation | | | | | | | | | | |

| **SUPPL TABLE S3 \|** Characteristics of clear cell renal cell cancer patients overall and by promoter methylation of *GREM1-ii, LAD1-ii, NEFH-ii, NEURL-ii* and *GATA5* in the hospital-based series | | | | | | |
| --- | --- | --- | --- | --- | --- | --- |
|  | **All ccRCC**  **n=64** | **Methylated *GREM 1-ii***  **n=9 (17.6%)** | **Methylated *LAD1-ii***  **n=17 (29.8%)** | **Methylated**  ***NEFH-ii***  **n=39 (72.2%)** | **Methylated *NEURL-ii***  **n=15 (27.3%)** | **Methylated *GATA5***  **n=14 (25.0%)** |
| Gender – no. (%) |  |  |  |  |  |  |
| Male | 39(60.9) | 7 (77.8) | 11 (64.7) | 28 (71.8) | 11 (73.3) | 9 (64.3) |
| Female | 25(39.1) | 2 (22.2) | 6 (35.3) | 11 (28.2) | 4 (26.7) | 5 (35.7) |
| *P*-value^a^ (U versus M) |  | 0.203 | 0.874 | 0.017 | 0.360 | >0.999 |
| Age – mean (SD) | 59.3 (12.1) | 61 (6.12) | 63.2 (12.2) | 61.2 (10.9) | 60.5 (10.0) | 63.4 (9.5) |
| *P*-value (U versus M) |  | 0.989 | 0.188 | 0.242 | 0.841 | 0.271 |
| Tumor size in mm – mean (SD) | 66.2 (34.9) | 65.3 (24.7) | 67.2 (30.7) | 64.7 (30.1) | 58.3 (24.2) | 67.4 (32) |
| *P*-value (U versus M) |  | 0.917 | 0.843 | 0.714 | 0.448 | 0.765 |
| Fuhrman grade – no. (%) |  |  |  |  |  |  |
| G1 | 2 (3.1) | 0 (0.0) | 1 (5.9) | 1 (2.6) | 1 (6.7) | 1 (7.1) |
| G2 | 27 (42.2) | 3 (33.3) | 4 (23.5) | 13 (33.3) | 6 (40.0) | 4 (28.6) |
| G3 | 28 (43.8) | 5 (55.6) | 10 (58.8) | 20 (51.3) | 7 (46.7) | 7 (50.0) |
| G4 | 7 (10.9) | 1 (11.1) | 2 (11.8) | 5 (12.8) | 1 (6.7) | 2 (14.3) |
| *P*-value (U versus M) |  | 0.886 | 0.134 | 0.088 | 0.345 | 0.238 |
| T – no. (%) |  |  |  |  |  |  |
| 1 | 35 (54.7) | 5 (55.6) | 8 (47.1) | 22 (56.4) | 10 (66.7) | 7 (50.0) |
| 2 | 10 (15.6) | 1 (11.1) | 5 (29.4) | 6 (15.4) | 2 (13.3) | 2 (14.3) |
| 3 | 18 (28.1) | 3 (33.3) | 4 (23.5) | 10 (25.6) | 3 (20.0) | 4 (28.6) |
| 4 | 1 (1.6) | 0 (0.0) | 0 (0.0) | 1 (2.6) | 0 (0.0) | 1 (7.1) |
| *P*-value (U versus M) |  | 0.945 | 0.169 | 0.716 | 0.827 | 0.363 |
| TNM stage^b^ – no. (%) |  |  |  |  |  |  |
| I | 2 (3.2) | 0 (0.0) | 1 (5.9) | 1 (2.6) | 0 (0.0) | 0 (0.0) |
| II | 39 (61.9) | 6 (66.7) | 12 (70.6) | 24 (63.2) | 11 (73.3) | 9 (64.3) |
| III | 10 (15.9) | 1 (11.1) | 3 (17.6) | 6 (15.8) | 1 (6.7) | 3 (21.4) |
| IV | 12 (19) | 2 (22.2) | 1 (5.9) | 7 (18.4) | 3 (20.0) | 2 (14.3) |
| Not available | 1 (1.6) | 0 (0.0) | 0 (0.0) | 1 (0.03) | 0 (0.0) | 0 (0.0) |
| *P*-value (U versus M) |  | 0.903 | 0.210 | 0.694 | 0.757 | 0.795 |
| ^a^ *P*-value tested with two-sample *t* test (for continuous variables) or χ^2^ test (for categorical variables); ^b^ TNM stage as defined in 1987; ^c^ HR from univariate Cox regression. U: unmethylated; M: methylated; SD: standard deviation. | | | | | | |

| **SUPPL TABLE S4 \|** Characteristics of clear cell renal cell cancer patients overall and by promoter methylation of *GREM1-ii, LAD1-ii, NEFH-ii, NEURL-ii, and GATA5* in the TCGA series | | | | | | |
| --- | --- | --- | --- | --- | --- | --- |
|  | **All ccRCC**  **n=232** | **Methylated *GREM1-ii***  **n=25 (11%)** | **Methylated**  ***LAD1-ii***  **n=33 (14%)** | **Methylated**  ***NEFH-ii***  **n=113 (49%)** | **Methylated**  ***NEURL-ii***  **n=220 (95%)** | **Methylated *GATA5***  **n=12 (5%)** |
| Gender – no. (%) |  |  |  |  |  |  |
| Male | 151 (65.1) | 21 (84.0) | 24 (72.7) | 77 (68.1) | 144 (65.5) | 8 (66.7) |
| Female | 81 (34.9) | 4 (16.0) | 9 (27.3) | 36 (31.9) | 76 (34.5) | 4 (33.3) |
| Age – mean (SD) | 61.7(12.3) | 64.9 (8.9) | 64.0 (9.9) | 62.6 (12.4) | 61.8 (12.5) | 66.1 (7.9) |
| Tumor size in mm – mean (SD) | 60.2 (36.7) | 97.2 (44.7) | 65.2 (35.8) | 69.1 (41.4) | 59.6 (36.8) | 77.3 (49.0) |
| Fuhrman grade – no. (%) |  |  |  |  |  |  |
| G1 | 5 (2.2) | 6 (24.0) | 10 (30.3) | 4 (3.5) | 5 (2.3) | 4 (33.3) |
| G2 | 108 (46.6) | 10 (40.0) | 16 (48.5) | 42 (37.2) | 103 (46.8) | 3 (25.0) |
| G3 | 88 (37.9) | 9 (36.0) | 6 (18.2) | 47 (41.6) | 82 (37.3) | 4 (33.3) |
| G4 | 28 (12.1) | 0 (0.0) | 1 (3.0) | 19 (16.8) | 28 (12.7) | 1 (8.3) |
| Not available | 3 (1.3) | 0 (0.0) | 0 (0.0) | 1 (0.9) | 2 (0.9) | 0 (0.0) |
| T – no. (%) |  |  |  |  |  |  |
| 1 | 130 (56.0) | 5 (20.0) | 11 (33.3) | 48 (42.5) | 124 (56.4) | 3 (25.0) |
| 2 | 28 (12.1) | 3 (12.0) | 3 (9.1) | 14 (12.4) | 25 (11.4) | 1 (8.3) |
| 3 | 71 (30.6) | 16 (64.0) | 19 (57.6) | 50 (44.3) | 68 (30.9) | 8 (66.7) |
| 4 | 3 (1.3) | 1 (4.0) | 0 (0.0) | 1 (0.9) | 3 (1.4) | 0 (0.0) |
| Not available | 0 (0.0) | 0 (0.0) | 0 (0.0) | 0 (0.0) | 0 (0.0) | 0 (0.0) |
| N – no. (%) |  |  |  |  |  |  |
| 0 | 104 (44.8) | 9 (36.0) | 14 (42.4) | 51 (45.1) | 100 (45.5) | 6 (50.0) |
| 1 | 7 (3.0) | 2 (8.0) | 1 (3.1) | 7 (6.2) | 7 (3.2) | 2 (16.7) |
| X | 121 (52.2) | 14 (56.0) | 18 (54.6) | 55 (48.7) | 113 (51.4) | 4 (33.3) |
| Not available | 0 (0.0) | 0 (0.0) | 0 (0.0) | 0 (0.0) | 0 (0.0) | 0 (0.0) |
| TNM stage – no. (%) |  |  |  |  |  |  |
| I | 130 (56.0) | 5 (20.0) | 11 (33.3) | 48 (42.5) | 124 (56.4) | 3 (25.0) |
| II | 27 (11.6) | 3 (12.0) | 3 (9.1) | 13 (11.5) | 24 (10.9) | 1 (8.3) |
| III | 71 (30.6) | 16 (64.0) | 19 (57.6) | 50 (44.2) | 68 (30.9) | 8 (66.7) |
| IV | 4 (1.7) | 1 (4.0) | 0 (0.0) | 2 (1.8) | 4 (1.8) | 0 (0.0) |
| Not available | 0 (0.0) | 0 (0.0) | 0 (0.0) | 0 (0.0) | 0 (0.0) | 0 (0.0) |
| ccRCC: clear cell renal cell carcinoma; SD: standard deviation | | | | | | |

| **SUPPL TABLE S5 \|** Genomic information of the selected probes from The Cancer Genome Atlas (TCGA) data for each of the five genes in the prognostic model | | | | |
| --- | --- | --- | --- | --- |
| **Gene** | **Probe ID** | **Chromosome** | **Genomic location** | **Distance from MSP primer** |
| *GREM1-ii* | cg10429922 | 15 | 33010143 | 81bp |
|  | cg08495115 | 15 | 33010399 | 338bp |
| *LAD1-ii* | cg07561547 | 1 | 201368783 | Within inside antisense primer |
|  | cg21964551 | 1 | 201368791 | Within inside amplicon |
|  | cg27425612 | 1 | 201368843 | Within outside sense primer |
| *NEFH-ii* | cg18129621 | 22 | 29875991 | 13bp |
|  | cg16042149 | 22 | 29875947 | 57bp |
| *NEURL-ii* | cg14233355 | 10 | 105253342 | Within outside sense primer |
|  | cg02324920 | 10 | 105253152 | 202 bp |
| *GATA5* | cg14980983 | 20 | 61051039 | Within inside amplicon |
|  | cg02484469 | 20 | 61051036 | Within inside amplicon |
|  | cg20265733 | 20 | 61051032 | Within inside amplicon |
|  | cg13845982 | 20 | 61051029 | Within inside amplicon |
|  | cg12664464 | 20 | 61051021 | Within inside antisense primer |
|  | cg16919517 | 20 | 61050914 | 85bp |

| **SUPPL TABLE S6 \|** Outside PCR mix preparation protocol. The reaction mix is an example of a multiplex nested MSP for 5 different genes (0.25µl primer per gene). | |
| --- | --- |
| Volume | Material |
| 14.65 μl | H2O |
| 2.5 μl | 10X buffer |
| 1.25 μl | dNTPs (5mM) |
| 1.25 μl | Flank sense primer mix (20 μM ) 5X0.25µl per gene |
| 1.25 μl | Flank antisense primer mix (20 μM ) 5X0.25µl per gene |
| 0.1 μl | Taq polymerase (5U/ μl) |
| 4 μl | Bisulfite treated DNA |
| 10X buffer: 166 mM (NH4)2SO4, 670 mM Tris pH 8.8, 67 mM MgCl2 * 6H2O, 100 mM β-mercapto-ethanol | |

| **SUPPL TABLE S7 \|** Outside PCR program | |  |
| --- | --- | --- |
| Time | Temperature | Cycles |
| 3 minutes | 95 ˚C |  |
| 30 seconds | 95 ˚C | 35X |
| 30 seconds | 56 ˚C |  |
| 30 seconds | 72 ˚C |  |
| 4 minutes | 72 ˚C |  |
| PCR products were diluted 1:1000 in 0.1x TE buffer for inside PCR | | |

| **SUPPL TABLE S8 \|** Inside PCR mix preparation protocol | |
| --- | --- |
| Volume | Material |
| 16.5 μl | H2O |
| 2.5 μl | 10X buffer |
| 0.25 μl | dNTPs (5mM) |
| 0.5 μl | sense primer (20 μM) |
| 0.5 μl | antisense primer (20μM) |
| 0.1 μl | Taq polymerase (5U/ μl) |
| 0.5 μl | Glycerol 50% |
| 4 μl | Diluted outside PCR product |
| 10X buffer: 166 mM (NH4)2SO4, 670 mM Tris pH 8.8, 67 mM MgCl2 * 6H2O, 100 mM β-mercapto-ethanol | |

| **SUPPL TABLE S9 \|** Inside PCR program | |  |
| --- | --- | --- |
| Time | Temperature | Cycles |
| 10 minutes | 95 ˚C |  |
| 30 seconds | 95 ˚C |  |
| 30 seconds | Varies per primer* | Varies per primer* |
| 30 seconds | 72 ˚C |  |
| 4 minutes | 72 ˚C |  |
| * Annealing temperature and numbers of cycles are dependent on the primers used. For exact temperature and number of cycles for each primer set, see SUPPL TABLE S10 | | |

| **SUPPL TABLE S10 \|** MSP primers for promoter methylation analysis of the 11 candidate genes | | | | | |
| --- | --- | --- | --- | --- | --- |
| **Gene** |  | **Sense primer** | **Antisense primer** | **T (°C)** | **PCR cycles** |
| GREM1  region I | Flank | TGGYGATAGGTTTTTGTTGG | CCCCTCTAAAAAAAACRAAACTA | 56 | 35 |
|  | U | GTTTTTGTTGGTGGTTAGTGAGTTT | AACAAAACTACAAAATACTCTAATCAAACA | 62 | 30 |
|  | M | TGTTGGCGGTTAGCGAGTTC | GAAACTACGAAATACTCTAATCGAACG | 62 | 30 |
| GREM1  region II | Flank | TTTTGGGGTGATTTTTTTTAGT | CACCCCTCCTACTTCCCC | 56 | 35 |
|  | U | GTGATTTTTTTTAGTTGGTTTGGGT | ACCCCTCCTACTTCCCCACA | 64 | 35 |
|  | M | ATTTTTTTTAGTCGGTTCGGGC | CCCTCCTACTTCCCCGCG | 64 | 35 |
| GREM  region III | Flank | GGGGTTTTTTTTGTTGAGGT | CTACTACCACCAACACCAAAAAC | 56 | 35 |
|  | U | TTTTTTTGTTGAGGTTGTGGATATT | CAAAAACATATTCAAAAACCTCCA | 65 | 25 |
|  | M | TTTGTTGAGGTCGCGGATATC | AAAACGTATTCGAAAACCTCCG | 65 | 25 |
| RASSF1A | Flank | GTTTAGTTTGGATTTTGGGGGAG | CCCRCAACTCAATAAACTCAAACTC | 56 | 35 |
|  | U | GGGGTTTGTTTTGTGGTTTTGTTT | AACATAACCCAATTAAACCCATACTTCA | 64 | 30 |
|  | M | GGGTTCGTTTTGTGGTTTCGTTC | TAACCCGATTAAACCCGTACTTCG | 64 | 30 |
| GATA5 | Flank | GGGGGTTGYGGAGTTTTA | CTAAAACACTCACCAACRAACA | 56 | 35 |
|  | U | AGTTTTAGTGGTTTGGGTTTTTAGTTTTT | CAAAAAAAACAACAAAACACAACAATAACA | 66 | 35 |
|  | M | GCGGTTCGGGTTTTTAGTTTTC | ACGACAAAACACGACGATAACG | 66 | 35 |
| LAD1  region I | Flank | ATTGGTTTTGAGTAGGAAGTTTTT | CCRCATTAATTACAACCCAAA | 56 | 35 |
|  | U | GAGTAGGAAGTTTTTTGTTTTTGTTTT | AACCCAAACCAACCCAACCA | 63 | 35 |
|  | M | GTAGGAAGTTTTTCGTTTTCGTTTC | CCAAACCGACCCGACCG | 63 | 35 |
| LAD1  region II | Flank | GTAGGGGTTTTYGTTTTTAGTTTAG | RAAAAACTTCCTACTCAAAACCAAT | 56 | 35 |
|  | U | TTAGTTTAGTTTTTTGGTATTTTGGGTTGT | TCAAAACCAATCCAACCAACTTAACA | 64 | 35 |
|  | M | TTTTTCGGTATTTCGGGTCGC | AAACCAATCCGACCAACTTAACG | 64 | 35 |
| NEFH  region I | Flank | TTTATTGTTAAGGGGTTGGATT | CRAAACTCATCATAACTAAACAAATAC | 56 | 35 |
|  | U | GTTAAGGGGTTGGATTTGGTTGT | CATCATAACTAAACAAATACACAAAACCA | 62 | 35 |
|  | M | GGGGTTGGATTCGGTCGC | AACTAAACAAATACGCGAAACCG | 62 | 35 |
| NEFH  region II | Flank | TYGATTTYGAGGGTTTTTTTTGA | CTCCTCCCCTACRACCC | 56 | 35 |
|  | U | GATTTTGAGGGTTTTTTTTGATGTAGTGTT | CCCCAACCAACCAACTCCA | 60 | 35 |
|  | M | GAGGGTTTTTTTTGACGTAGCGTC | CCGACCGACCGACTCCG | 60 | 35 |
| SCUBE3 | Flank | GYGGAGTTTGTATTGATTTATTGATT | CCRCTACCTACAAAAATTTACAATC | 56 | 35 |
|  | U | GGAGTTTGTATTGATTTATTGATTGTGTGT | CCTACAAAAATTTACAATCCAACAATAAAAAAACA | 60 | 35 |
|  | M | TTGTATTGATTTATTGATTGCGCGC | AAAAATTTACAATCCGACGATAAAAAAACG | 60 | 35 |
| PCDH8 region I | Flank | TAGTATATYGGYGGTTATAAAGGTAAA | AATAACAACTCCRAACCTCCAA | 56 | 35 |
|  | U | GGTTATAAAGGTAAAGGTGGTGTGAGGT | AACCTCCAACAAACTCTAAAAACACACA | 60 | 35 |
|  | M | TAAAGGCGGCGCGAGGC | CCAACGAACTCTAAAAACGCGCG | 60 | 35 |
| PCDH8 region II | Flank | TGGAGTTGGTGGTTTAGGA | TCTTCCRCCAACTCCACT | 56 | 35 |
|  | U | TTGGTGGTTTAGGATGGTGGTT | CAACTCCACTTCAACCACAACA | 60 | 35 |
|  | M | TGGTTTAGGACGGCGGTC | TCCACTTCGACCACGACG | 60 | 35 |
| SFRP1 | Flank | TTTAGTTTTGTAGTTTTYGGAGTTAG | CCCCRACCAATAACRACCCTC | 56 | 35 |
|  | U | GTTTTGTAGTTTTTGGAGTTAGTGTTGTGT | CTCAACCTACAATCAAAAACAACACAAACA | 66 | 25 |
|  | M | TGTAGTTTTCGGAGTTAGTGTCGCGC | CCTACGATCGAAAACGACGCGAACG | 66 | 25 |
| BNC1 | Flank | TTGYGGATTTTGGAGTTTTTGT | TTCCTATCACTCCAAAAAAACCT | 56 | 35 |
|  | U | GGATTTTGGAGTTTTTGTTGTGGTTTT | CACTCCAAAAAAACCTCAAAACAACA | 66 | 35 |
|  | M | TGGAGTTTTTGTCGCGGTTTC | CCAAAAAAACCTCGAAACGACG | 66 | 35 |
| NEURL region I | Flank | GGTTTAGGGTTTTGTTTGTGG | CCTCAACTTCCTTTCTCTAAACTT | 56 | 35 |
|  | U | TTGTTTGTGGTTTTTGTTTTTGTTAT | CTTTCTCTAAACTTCACAATCCTTAACA | 65 | 35 |
|  | M | GTTTGTGGTTTTCGTTTTCGTTAC | TCTAAACTTCGCGATCCTTAACG | 65 | 35 |
| NEURL region II | Flank | TAAGGTTAGTTTYGGGTGTTATTATATT | RCCCACTAAACCAAATATAACCC | 56 | 35 |
|  | U | GTGTTATTATATTTGTTTGATGTTTTATTTTGATTTGT | ACTAAACCAAATATAACCCCAAAACACA | 64 | 35 |
|  | M | TTCGTTTGATGTTTTATTTCGATTCGC | CCAAATATAACCCCGAAACGCG | 64 | 35 |
| CDO1 | Flank | GGGAGAGATTGYGYGGAGTTTA | AAAACCACCCAAAAAAAATAAC | 56 | 35 |
|  | U | GATTTTTGGGATGTTGGAGATAAT | AAAACAAAAAAACCCTACAAACACA | 64 | 30 |
|  | M | TTTTTGGGACGTCGGAGATAAC | CGAAAAAACCCTACGAACACG | 64 | 30 |
| U: unmethylated; M: methylated; T: temperature | | | | | |

| **SUPPL TABLE S11 \|** Associations of clinicopathological characteristics and methylation markers with ccRCC-specific survival in the population-based series | | | | | | | | | |
| --- | --- | --- | --- | --- | --- | --- | --- | --- | --- |
|  |  | **Univariable analysis** | | | | **Multivariate analysis^a^** | | | |
|  |  | **HR** | **95% CI** | | ***P*-value** | **HR** | **95% CI** | ***P*-value** | |
| Current prognostic variables | | | | | | | | | |
| Gender | Male | 1.0 (ref) |  | |  | 1.0 (ref) |  |  | |
|  | Female | 0.87 | 0.59-1.28 | | 0.47 | 1.03 | 0.68-1.57 | 0.89 | |
| Age at diagnosis | Continuous | 1.01 | 0.98-1.04 | | 0.67 | 1.01 | 0.98-1.05 | 0.43 | |
| TNM stage^b^ | I | 1.0 (ref) |  | |  | 1.0 (ref) |  |  | |
|  | II | 1.86 | 0.58-5.97 | | 0.30 | 1.58 | 0.48-5.22 | 0.45 | |
|  | III | **3.79** | **1.18-12.19** | | **0.03** | 2.64 | 0.75-9.25 | 0.13 | |
|  | IV | **10.27** | **2.72-38.85** | | **0.001** | **7.13** | **1.65-30.77** | **0.01** | |
| Fuhrman grade | G1 | 1.0 (ref) |  | |  | 1.0 (ref) |  |  | |
|  | G2 | 1.06 | 0.57-1.99 | | 0.85 | 1.02 | 0.52-1.99 | 0.96 | |
|  | G3 | 1.44 | 0.76-2.73 | | 0.27 | 1.37 | 0.69-2.72 | 0.36 | |
|  | G4 | **3.09** | **1.57-6.10** | | **0.001** | **2.77** | **1.33-5.76** | **0.01** | |
| Tumor size | Continuous | **1.01** | **1.01-1.02** | | **<0.001** | 1.00 | 0.99-1.01 | 0.55 | |
| Potential prognostic methylation markers, methylated vs. unmethylated (% methylation)^c^ | | | | | | | | | |
| *GREM1-i* | 83/284 (29%) | **1.93** | **1.26-2.95** | **0.003** | | **1.86** | **1.16-2.97** | | **0.01** |
| *GREM1-ii* | 162/298 (42%) | 0.89 | 0.59-1.33 | 0.56 | | 0.87 | 0.56-1.36 | | 0.54 |
| *GREM1-iii* | 125/314 (40%) | 1.29 | 0.87-1.91 | 0.21 | | 1.24 | 0.81-1.90 | | 0.31 |
| *LAD1-i* | 87/313 (28%) | **2.25** | **1.51-3.35** | **<0.0001** | | **2.26** | **1.47-3.48** | | **<0.0001** |
| *LAD1-ii* | 111/302 (37%) | **1.89** | **1.27-2.80** | **0.002** | | **1.71** | **1.12-2.61** | | **0.01** |
| *NEFH-i* | 81/290 (28%) | **1.76** | **1.16-2.67** | **0.007** | | **1.74** | **1.11-2.74** | | **0.02** |
| *NEFH-ii* | 190/277 (69%) | 1.00 | 0.65-1.56 | 0.97 | | 1.01 | 0.64-1.59 | | 0.98 |
| *NEURL-i* | 128/325 (39%) | 1.32 | 0.90-1.95 | 0.15 | | 1.34 | 0.89-2.02 | | 0.16 |
| *NEURL-ii* | 101/288 (35%) | **1.99** | **1.31-3.04** | **0.001** | | **1.94** | **1.21-3.13** | | **0.01** |
| *PCDH8-i* | 230/291 (79%) | 1.56 | 0.90-2.70 | 0.12 | | 1.83 | 0.96-3.50 | | 0.07 |
| *PCDH8-ii* | 243/299 (81%) | 0.73 | 0.46-1.17 | 0.19 | | 0.77 | 0.47-1.27 | | 0.31 |
| *SCUBE3* | 234/292 (80%) | 0.96 | 0.59-1.58 | 0.88 | | 1.00 | 0.60-1.68 | | 0.99 |
| *BNC1* | 158/291 (54%) | 1.21 | 0.80-1.83 | 0.37 | | 1.20 | 0.78-1.86 | | 0.40 |
| *CDO1* | 118/306 (39%) | 1.49 | 0.99-2.23 | 0.06 | | 1.43 | 0.92-2.23 | | 0.11 |
| *GATA5* | 96/293 (33%) | **1.69** | **1.12-2.56** | **0.01** | | **1.69** | **1.09-2.62** | | **0.02** |
| *RASSF1A* | 351/300 (84%) | 0.93 | 0.55-1.58 | 0.80 | | 0.98 | 0.56-1.71 | | 0.94 |
| *SFRP1* | 57/315 (18%) | **2.25** | **1.44-3.51** | **<0.0001** | | **1.89** | **1.16-3.08** | | **0.01** |
| ^a^ Adjusted for age at diagnosis, gender, Fuhrman grade, tumor size and TNM stage; ^b^ TNM stage as defined in 1987; ^c^ MSP failed to obtain results for all samples due to poor DNA quality and low quantity of some samples. HR: hazard ratio; 95% CI: 95% confidence interval | | | | | | | | | |

| **SUPPL TABLE S12 \|** DNA integrity and MSP results for a subset of samples from the population-based series | | | | | | | | | | | |
| --- | --- | --- | --- | --- | --- | --- | --- | --- | --- | --- | --- |
| **Sample** | **100 bp** | **200 bp** | **300 bp** | **400 bp** | **500 bp** | **600 bp** | **NEFH_2** | **GREM2** | **GATA5** | **NEURL2** | **LAD2** |
| 0001T | x | x |  |  |  |  | Failed | U | U | U | U |
| 0006T | x | x | x |  |  |  | Failed | U | U | U | U |
| 0007T | x | x | x |  |  |  | M | M | U | U | M |
| 0009T | x | x | x |  |  |  | M | M | M | M | M |
| 0011T | x | x | x |  |  |  | Failed | M | Failed | M | M |
| 0014T | x | x | x |  |  |  | M | U | U | M | Failed |
| 0016T |  |  |  |  |  |  | Failed | U | U | Failed | M |
| 0029T | x | x |  |  |  |  | U | U | U | U | Failed |
| 0033T |  |  |  |  |  |  | U | U | U | U | U |
| 0036T | x | x | x | x |  |  | M | U | U | U | Failed |
| 0038T | x | x | x | x |  |  | M | U | U | M | M |
| 0048T |  |  |  |  |  |  | M | M | U | M | U |
| 0049T |  |  |  |  |  |  | U | U | U | U | U |
| 0051T |  |  |  |  |  |  | M | U | U | M | U |
| 0054T | x | x | x | x |  |  | M | U | U | U | U |
| 0073T | x | x | x | x |  |  | M | U | U | M | Failed |
| 0115T | x | x |  |  |  |  | Failed | U | U | U | faifed |
| 0126T |  |  |  |  |  |  | U | U | U | U | U |
| 0161T | x | x | x | x |  |  | M | U | U | U | Failed |
| 0166T | x | x | x |  |  |  | M | M | M | M | M |
| 0167T |  |  |  |  |  |  | U | U | Failed | Failed | M |
| 0170T |  |  |  |  |  |  | U | U | Failed | U | U |
| 0174T | x | x | x | x | x |  | M | U | U | M | U |
| 0176T | x | x | x | x |  |  | U | U | U | U | U |
| 0180T | x | x | x |  |  |  | M | U | U | U | U |
| 0183T | x | x | x | x | x |  | M | U | M | M | M |
| 0185T | x | x | x |  |  |  | U | U | U | U | U |
| 0187T |  |  |  |  |  |  | U | U | U | U | U |
| 0189T | x | x | x |  |  |  | M | M | M | M | M |
| 0191T | x | x | x | x |  |  | M | M | U | U | U |
| 0192T | x | x | x |  |  |  | M | U | U | U | M |
| 0225T | x | x | x |  |  |  | M | M | U | U | M |
| 0226T | x | x | x |  |  |  | M | M | U | U | M |
| 0232T |  |  |  |  |  |  | M | M | M | U | Failed |
| 0234T | x | x | x | x | x |  | M | M | U | U | M |
| 0237T | x | x | x | x |  |  | M | M | M | U | U |
| 0257T | x | x | x | x |  |  | M | U | U | U | M |
| 0260T | x | x | x |  |  |  | U | U | U | U | U |
| 0261T | x | x | x | x |  |  | Failed | U | U | U | M |
| 0262T | x | x | x | x |  |  | U | U | U | U | Failed |
| 0263T | x | x | x |  |  |  | M | U | U | U | U |
| 0287T | x | x | x | x |  |  | M | M | M | U | U |
| 0302T | x | x | x | x |  |  | M | M | U | U | U |
| 0304T | x | x | x | x | x |  | U | M | U | U | U |
| 0309T | x | x | x |  |  |  | M | U | U | U | U |
| 0332T |  |  |  |  |  |  | U | U | U | U | U |
| 0334T | x | x | x | x |  |  | M | M | U | U | M |
| 0336T | x | x |  |  |  |  | M | U | U | M | U |
| 0338T | x | x |  |  |  |  | M | U | U | U | M |
| 0342T | x | x |  |  |  |  | U | M | U | U | U |
| 0343T |  |  |  |  |  |  | U | Failed | Failed | Failed | Failed |
| 0344T | x | x |  |  |  |  | M | U | U | M | U |
| 0347T |  |  |  |  |  |  | U | U | U | U | U |
| 0349T |  |  |  |  |  |  | U | U | Failed | Failed | U |
| 0371T | x | x | x | x |  |  | M | M | U | U | U |
| 0372T | x | x |  |  |  |  | U | U | Failed | U | U |
| 0392T | x | x | x |  |  |  | U | U | U | U | U |
| 0394T | x | x | x | x |  |  | M | M | U | U | M |
| 0397T | x | x | x | x |  |  | M | M | U | U | M |
| 0398T | x | x | x | x |  |  | M | Failed | U | U | M |
| 0400T | x | x | x | x | x |  | M | M | U | U | M |
| 0406T | x | x | x |  |  |  | U | Failed | U | U | U |
| 0419T | x | x |  |  |  |  | U | M | U | U | U |
| 0422T | x | x |  |  |  |  | M | M | M | U | Failed |
| 0429T | x | x | x |  |  |  | M | U | U | U | U |
| 0452T | x | x |  |  |  |  | U | U | U | U | U |
| 0454T | x | x |  |  |  |  | M | U | U | U | U |
| 0459T |  |  |  |  |  |  | M | U | Failed | U | U |
| 0461T | x | x |  |  |  |  | U | U | U | U | U |
| 0463T |  |  |  |  |  |  | M | U | Failed | M | U |
| 0497T | x | x | x | x |  |  | M | M | U | U | U |
| 0502T | x | x | x | x |  |  | M | M | U | U | M |
| 0506T | x | x | x |  |  |  | Failed | U | U | U | U |
| 0559T | x | x | x |  |  |  | M | M | U | U | M |
| 0562T | x | x | x |  |  |  | U | U | U | U | U |
| 0564T | x | x | x |  |  |  | U | U | U | U | U |
| 0568T |  |  |  |  |  |  | M | M | U | M | M |
| 0592T | x | x | x |  |  |  | M | M | U | M | M |
| 0593T | x | x | x |  |  |  | Failed | U | U | U | M |
| 0596T |  |  |  |  |  |  | U | U | M | M | M |
| 0597T | x | x | x |  |  |  | U | M | U | U | M |
| 0641T | x | x | x |  |  |  | U | M | U | U | U |
| 0645T | x | x | x |  |  |  | U | M | M | U | M |
| 0647T | x | x | x |  |  |  | U | U | U | U | M |
| 0651T | x | x | x |  |  |  | U | M | M | M | M |
| 0669T |  |  |  |  |  |  | M | M | M | M | M |
| 0670T | x | x | x |  |  |  | M | U | U | U | U |
| 0672T |  |  |  |  |  |  | U | U | U | M | U |
| 0674T | x | x | x |  |  |  | Failed | U | U | U | U |
| 0676T | x | x | x |  |  |  | U | U | U | U | U |
| 0678T | x | x | x | x |  |  | U | U | U | M | U |
| 0679T | x | x | x |  |  |  | U | U | U | U | U |
| 0682T | x | x | x |  |  |  | Failed | M | M | U | U |
| 0683T | x | x |  |  |  |  | Failed | U | U | U | M |
| 0687T | x | x |  |  |  |  | U | U | U | U | M |
| 0689T | x | x |  |  |  |  | U | U | U | U | U |
| 0690T | x | x |  |  |  |  | Failed | M | M | M | M |
| 0692T |  |  |  |  |  |  | M | M | M | M | U |
| 0695T | x | x | x | x | x |  | U | M | M | M | M |
| 0696T | x | x |  |  |  |  | Failed | U | U | U | U |
| 0753T | x | x |  |  |  |  | U | U | M | U | U |
| 0755T | x | x | x |  |  |  | Failed | U | U | Failed | U |
| 0757T |  |  |  |  |  |  | Failed | U | U | U | U |
| 0777T | x | x | x |  |  |  | M | U | M | U | M |
| 0787T | x | x | x | x |  |  | U | M | M | U | M |
| 0788T | x | x |  |  |  |  | U | U | U | U | U |
| 0790T | x | x | x | x |  |  | Failed | U | U | Failed | U |
| 0792T | x | x | x | x |  |  | U | Failed | U | U | U |
| 0794T | x | x | x | x |  |  | Failed | U | M | M | U |
| 0796T | x | x | x |  |  |  | U | U | U | U | U |
| 0826T | x | x | x | x |  |  | U | U | U | U | M |
| 0827T | x | x | x | x |  |  | U | U | U | Failed | M |
| 0829T | x | x | x | x |  |  | U | M | M | Failed | M |
| 0854T | x | x | x | x |  |  | U | U | U | U | M |
| 0879T | x | x | x |  |  |  | U | U | U | Failed | M |
| 0882T |  |  |  |  |  |  | Failed | U | U | U | M |
| 0897T | x | x |  |  |  |  | U | U | U | U | M |
| 0904T | x | x | x |  |  |  | Failed | U | U | M | M |
| 0926T | x | x | x | x | x |  | U | M | M | M | M |
| 0938T | x | x | x | x |  |  | U | U | U | Failed | M |
| 0944T |  |  |  |  |  |  | U | U | U | U | M |
| 0947T | x | x |  |  |  |  | Failed | U | U | U | M |
| 0949T | x | x |  |  |  |  | U | U | M | Failed | M |
| 0952T | x | x |  |  |  |  | U | U | M | U | M |
| 0954T | x | x |  |  |  |  | U | M | M | M | M |
| 0960T | x | x | x |  |  |  | U | M | M | U | U |
| 0962T | x | x |  |  |  |  | M | M | M | M | M |
| 0965T | x | x | x |  |  |  | Failed | U | Failed | U | U |
| 0969T | x | x | x |  |  |  | U | U | U | U | U |
| 0973T |  |  |  |  |  |  | U | M | U | U | M |
| 0977T | x | x |  |  |  |  | U | M | M | M | U |
| 0979T | x | x |  |  |  |  | M | U | U | U | M |
| 0985T | x | x | x |  |  |  | M | M | M | Failed | M |
| 0989T |  |  |  |  |  |  | U | U | M | Failed | U |
| 0991T | x | x |  |  |  |  | U | U | Failed | M | U |
| 0996T |  |  |  |  |  |  | U | U | U | Failed | U |
| 1001T | x | x |  |  |  |  | U | U | U | Failed | M |
| 1003T | x | x | x |  |  |  | Failed | M | U | Failed | U |
| 1004T | x | x |  |  |  |  | Failed | U | Failed | Failed | M |
| 1010T | x | x | x | x |  |  | U | M | Failed | Failed | M |
| 1013T | x | x |  |  |  |  | U | M | Failed | U | U |
| 1016T | x | x |  |  |  |  | M | U | U | Failed | U |
| 1021T |  |  |  |  |  |  | M | U | U | Failed | U |
| 1164T | x | x | x |  |  |  | U | U | U | U | M |
| 1167T | x | x | x | x |  |  | M | M | U | Failed | U |
| 1170T | x | x | x | x |  |  | U | U | U | M | M |
| 1172T | x | x | x | x |  |  | U | M | M | Failed | M |
| 1297T | x | x |  |  |  |  | Failed | U | U | U | U |
| 1299T | x | x |  |  |  |  | U | U | U | M | U |
| 1303T | x | x |  |  |  |  | Failed | M | U | U | U |
| 1306T | x | x | x |  |  |  | M | M | U | M | U |
| 1311T | x | x | x |  |  |  | M | U | M | M | M |
| 1314T | x | x | x |  |  |  | U | U | M | M | M |
| 1317T | x | x | x |  |  |  | M | M | M | Failed | M |
| 1323T | x | x | x |  |  |  | Failed | M | U | M | M |
| 1325T | x | x | x |  |  |  | U | U | Failed | U | U |
| 1329T | x | x | x |  |  |  | Failed | U | Failed | Failed | U |
| 1332T | x | x | x |  |  |  | U | M | M | M | U |
| 1334T | x | x | x |  |  |  | M | U | U | U | U |
| 1336T | x | x | x | x |  |  | U | U | U | U | U |
| 1339T | x | x |  |  |  |  | M | M | U | Failed | U |
| 1342T |  |  |  |  |  |  | M | U | U | U | U |
| 1347T | x | x | x |  |  |  | M | U | M | U | U |
| 1350T | x | x | x |  |  |  | M | M | U | U | M |
| 1354T | x | x |  |  |  |  | M | U | U | U | U |
| 1499T | x | x |  |  |  |  | M | M | U | M | M |
| 1502T | x | x | x | x |  |  | M | M | U | M | U |
| 1519T | x | x | x | x |  |  | M | M | M | M | U |
| 1524T | x | x | x | x |  |  | M | M | M | M | M |
| 1615T | x | x | x |  |  |  | M | Failed | U | U | Failed |
| 1716T |  |  |  |  |  |  | M | U | U | U | M |
| 1718T | x | x | x | x | x |  | M | U | U | U | U |
| 1782T |  |  |  |  |  |  | M | U | U | U | U |
| 1787T |  |  |  |  |  |  | U | U | U | U | U |
| 1806T | x | x |  |  |  |  | M | U | M | U | M |
| 1809T |  |  |  |  |  |  | U | U | U | U | U |
| 1885T | x | x | x |  |  |  | M | U | U | U | M |
| 1907T | x | x | x | x |  |  | M | M | M | M | M |
| 1910T | x | x |  |  |  |  | M | M | M | M | M |
| 1912T | x | x | x |  |  |  | M | M | U | U | M |
| 1918T | x | x | x |  |  |  | M | M | U | M | M |
| 1919T | x | x | x |  |  |  | M | M | U | M | M |
| 2002T |  |  |  |  |  |  | M | M | U | M | M |
| 2035T |  |  |  |  |  |  | U | U | U | M | M |
| 2040T | x | x |  |  |  |  | M | M | U | U | U |
| 2043T |  |  |  |  |  |  | U | U | U | U | U |
| 2049T | x | x |  |  |  |  | M | M | M | M | M |
| 2057T | x | x |  |  |  |  | M | M | M | M | M |
| 2066T | x | x | x |  |  |  | M | M | M | U | M |
| 2074T | x | x | x |  |  |  | U | U | U | U | U |
| 2078T | x | x | x |  |  |  | Failed | M | U | U | M |
| 2086T |  |  |  |  |  |  | M | M | U | M | M |
| 2088T | x | x |  |  |  |  | U | Failed | U | U | U |
| 2092T |  |  |  |  |  |  | Failed | U | U | U | U |
| 2103T | x | x |  |  |  |  | Failed | U | U | U | M |
| 2108T |  |  |  |  |  |  | Failed | U | U | U | U |
| 2109T |  |  |  |  |  |  | U | Failed | U | U | U |
| 2120T |  |  |  |  |  |  | U | U | U | U | U |
| 2124T | x | x | x |  |  |  | Failed | M | M | M | M |
| 2134T |  |  |  |  |  |  | U | U | U | U | U |
| 2411T | x | x | x | x |  |  | U | M | M | M | M |
| 2414T | x | x | x | x |  |  | U | U | U | U | U |
| 2438T | x | x | x | x |  |  | U | U | M | U | M |
| 2446T | x | x | x | x |  |  | U | U | U | U | M |
| 2447T | x | x | x | x |  |  | Failed | U | U | U | U |
| 2449T | x | x | x | x | x |  | U | U | U | U | M |
| 2451T | x | x | x |  |  |  | U | U | M | U | M |
| 2455T |  |  |  |  |  |  | Failed | M | M | M | M |
| 2456T | x | x | x | x |  |  | M | M | M | M | M |
| 2458T | x | x | x | x |  |  | Failed | U | U | U | U |
| 2460T |  |  |  |  |  |  | M | Failed | M | U | M |
| 2470T | x | x | x |  |  |  | M | U | M | Failed | M |
| 2471T | x | x | x | x |  |  | U | U | U | U | U |
| G3002T | x | x | x | x | x |  | M | U | U | M | M |
| G3003T | x | x | x | x |  |  | M | U | U | M | U |
| G3004T | x | x | x | x | x |  | M | M | U | U | M |
| G3005T | x | x | x |  |  |  | M | U | U | U | U |
| G3006T | x | x | x | x |  |  | M | M | M | M | M |
| G3007T | x | x | x |  |  |  | M | M | U | Failed | U |
| G3008T | x | x | x |  |  |  | M | U | U | U | Failed |
| G3009T | x | x | x | x |  |  | M | U | Failed | U | U |
| G3010T | x | x | x | x |  |  | M | M | U | Failed | M |
| G3011T |  |  |  |  |  |  | M | U | U | U | U |
| G3012T |  |  |  |  |  |  | M | U | U | U | U |
| G3013T | x | x | x | x | x |  | M | M | U | M | M |
| G3014T | x | x | x | x | x |  | M | U | U | U | U |
| G3015T | x | x | x |  |  |  | M | U | U | U | U |
| G3016T |  |  |  |  |  |  | U | U | U | U | U |
| G3017T | x | x | x | x | x |  | M | M | U | U | U |
| G3018T | x | x |  |  |  |  | M | U | U | U | U |
| G3019T | x | x |  |  |  |  | M | U | U | U | M |
| G3022T | x | x | x | x |  |  | M | M | U | U | U |
| G3023T |  |  |  |  |  |  | M | U | M | U | M |
| G3024T | x | x |  |  |  |  | Failed | U | Failed | U | U |
| G3026T | x | x | x |  |  |  | U | U | U | Failed | U |
| G3027T | x | x | x |  |  |  | Failed | U | U | M | U |
| G3028T | x | x |  |  |  |  | M | M | M | M | M |
| G3029T | x | x | x | x |  |  | M | M | U | U | M |
| G3030T | x | x | x | x |  |  | M | Failed | U | U | U |
| G3031T | x | x | x | x |  |  | M | M | Failed | U | M |
| G3032T | x | x | x |  |  |  | M | U | M | M | U |
| G3035T | x | x | x | x |  |  | M | U | M | U | U |
| G3036T | x | x | x | x |  |  | M | M | U | U | U |
| G3037T | x | x | x |  |  |  | U | U | U | U | U |
| G3038T | x | x |  |  |  |  | U | U | U | U | U |
| G3040T | x | x | x |  |  |  | M | U | Failed | M | M |
| G3041T | x | x | x | x |  |  | U | U | U | U | U |
| G3042T | x | x | x | x |  |  | M | M | U | M | M |
| G3043T | x | x | x |  |  |  | M | M | U | U | U |
| G3045T |  |  |  |  |  |  | U | U | U | Failed | U |
| G3046T | x | x | x | x |  |  | M | M | Failed | M | U |
| G3047T | x | x | x | x |  |  | M | U | U | M | U |
| G3048T | x | x | x | x |  |  | M | M | U | U | U |
| G3049T |  |  |  |  |  |  | M | M | U | U | U |
| G3052T | x | x | x | x |  |  | U | U | U | U | U |
| G3053T | x | x | x | x | x |  | M | U | U | U | M |
| G3054T | x | x | x | x |  |  | M | U | M | M | U |
| G3055T |  |  |  |  |  |  | U | U | U | U | U |
| G3056T | x | x | x | x |  |  | M | U | U | U | U |
| G3057T | x | x | x | x |  |  | M | M | M | U | U |
| G3058T | x | x | x | x |  |  | M | U | M | U | U |
| G3060T | x | x | x |  |  |  | M | U | U | U | M |
| G3061T | x | x | x |  |  |  | M | M | U | U | U |
| G3062T | x | x | x |  |  |  | M | U | U | U | U |
| G3063T | x | x | x |  |  |  | M | U | U | U | M |
| G3064T | x | x |  |  |  |  | M | M | U | U | U |
| G3065T | x | x | x | x | x |  | M | M | M | M | U |
| G3067T | x | x | x |  |  |  | M | U | U | U | U |
| G3068T | x | x |  |  |  |  | M | M | U | U | M |
| G3069T | x | x |  |  |  |  | M | U | U | U | U |
| G3070T | x | x | x |  |  |  | M | M | U | U | U |
| G3071T | x | x | x | x | x |  | M | U | U | U | U |
| G3072T | x | x | x | x | x |  | U | U | U | U | U |
| G3074T | x | x | x | x |  |  | U | U | U | U | U |
| G3075T | x | x | x | x | x |  | M | M | M | U | U |
| G3076T | x | x | x | x |  |  | M | M | U | U | U |
| G3077T | x | x | x | x |  |  | U | U | U | U | U |
| G3079T | x | x | x | x |  |  | M | M | U | M | U |
| G3080T | x | x | x | x |  |  | M | M | U | M | M |
| G3081T | x | x | x | x |  |  | U | U | U | Failed | U |
| G3082T | x | x | x | x |  |  | M | M | U | U | U |
| G3083T | x | x | x | x |  |  | M | U | U | U | U |
| G3084T | x | x | x | x |  |  | M | U | U | U | U |
| G3085T | x | x |  |  |  |  | Failed | U | U | U | U |
| G3086T | x | x |  |  |  |  | U | U | U | U | M |
| G3088T |  |  |  |  |  |  | U | M | U | U | U |
| G3089T | x | x | x | x |  |  | M | M | M | U | M |
| G3090T | x | x |  |  |  |  | M | M | M | M | M |
| G3091T | x | x |  |  |  |  | M | U | M | M | U |
| G3092T | x | x | x |  |  |  | U | U | U | U | U |
| G3093T | x | x | x | x |  |  | U | Failed | M | U | M |
| G3094T | x | x | x |  |  |  | U | Failed | U | U | M |
| G3095T | x | x | x |  |  |  | M | U | U | U | U |
| G3096T | x | x |  |  |  |  | U | U | M | U | U |
| G3097T | x | x |  |  |  |  | U | U | U | M | M |
| G3098T | x | x | x | x |  |  | U | U | M | U | U |
| G3099T | x | x |  |  |  |  | U | U | U | U | U |
| G3100T | x | x | x | x |  |  | M | U | U | U | U |
| G3101T | x | x | x |  |  |  | M | M | U | M | U |
| G3102T | x | x | x |  |  |  | M | M | M | M | U |
| G3103T | x | x |  |  |  |  | U | U | U | U | U |
| G3104T | x | x |  |  |  |  | M | M | U | U | U |
| G3106T | x | x | x |  |  |  | U | Failed | U | U | U |
| G3107T | x | x |  |  |  |  | M | U | M | M | M |
| G3108T1 | x | x | x | x |  |  | M | U | U | U | U |
| G3109T | x | x | x | x | x |  | M | M | M | M | U |
| G3110T |  |  |  |  |  |  | M | U | M | M | M |
| G3111T | x | x | x |  |  |  | M | U | U | U | M |
| G3113T | x | x | x |  |  |  | U | M | U | U | M |
| G3114T | x | x |  |  |  |  | U | Failed | U | U | U |
| G3115T | x | x | x | x | x |  | U | M | U | U | U |
| G3116T | x | x | x | x |  |  | M | Failed | U | U | U |
| G3119T | x | x | x | x |  |  | M | U | M | U | U |
| G3120T | x | x | x | x |  |  | M | U | U | U | U |
| G3121T | x | x | x |  |  |  | U | Failed | U | M | U |
| G3122T | x | x | x |  |  |  | M | M | M | M | M |
| G3123T | x | x | x | x |  |  | M | Failed | M | U | U |
| G3124T | x | x | x | x |  |  | U | U | U | U | U |
| G3125T | x | x | x | x |  |  | M | M | M | U | U |
| G3126T | x | x | x |  |  |  | M | U | U | U | U |
| G3127T | x | x | x | x |  |  | M | U | M | U | U |
| G3128T | x | x | x | x |  |  | U | U | U | U | U |
| G3129T | x | x |  |  |  |  | M | U | M | M | U |
| G3130T | x | x | x | x |  |  | M | M | M | U | U |
| G3131T1 | x | x | x |  |  |  | M | M | M | Failed | M |
| G3131T2 | x | x | x | x |  |  | U | U | U | U | U |
| G3132T | x | x | x |  |  |  | U | U | U | U | U |
| G3133T | x | x | x | x |  |  | U | U | U | M | M |
| G3134T | x | x | x | x |  |  | U | U | U | U | U |
| G3135T | x | x | x | x |  |  | Failed | M | U | U | U |
| G3136T | x | x | x | x |  |  | M | M | U | M | M |
| G3139T | x | x | x | x |  |  | U | M | U | U | U |
| G3140T | x | x |  |  |  |  | U | M | M | M | U |
| G3141T | x | x |  |  |  |  | U | U | U | U | M |
| G3142T |  |  |  |  |  |  | M | M | U | U | U |
| G3143T | x | x | x | x |  |  | M | M | U | U | U |
| G3144T | x | x |  |  |  |  | M | U | M | U | M |
| G3145T | x | x | x |  |  |  | M | U | U | U | U |
| G3146T | x | x |  |  |  |  | U | U | U | U | U |
| G3147T | x | x | x | x |  |  | U | U | U | U | U |
| G3148T | x | x |  |  |  |  | Failed | M | U | M | M |
| G3149T | x | x | x |  |  |  | Failed | M | M | M | U |
| G3150T | x | x | x | x |  |  | U | M | U | U | U |
| G3151T | x | x | x | x |  |  | M | U | U | U | U |
| G3152T | x | x |  |  |  |  | U | M | U | U | Failed |
| G3155T | x | x | x |  |  |  | Failed | U | U | U | U |
| G3156T | x | x | x |  |  |  | Failed | U | U | U | U |
| G3157T | x | x | x | x |  |  | M | M | M | U | M |
| G3158T | x | x | x | x |  |  | M | U | M | M | M |
| G3159T | x | x | x | x |  |  | M | U | U | U | M |
| G3160T | x | x | x |  |  |  | Failed | U | Failed | U | U |
| G3162T | x | x | x | x |  |  | M | U | U | U | U |
| G3163T | x | x | x | x |  |  | M | U | U | U | M |
| G3164T | x | x | x | x |  |  | M | U | U | U | U |
| G3165T | x | x | x | x |  |  | M | U | U | U | U |
| G3166T | x | x | x | x |  |  | Failed | U | U | U | U |
| G3167T | x | x | x | x |  |  | M | U | U | U | U |
| G3168T |  |  |  |  |  |  | U | U | U | U | U |
| G3169T |  |  |  |  |  |  | Failed | U | U | U | U |
| G3170T | x | x | x |  |  |  | M | U | U | U | M |
| G3171T | x | x | x | x |  |  | M | U | U | U | U |
| G3172T | x | x | x | x |  |  | U | M | U | U | U |
| G3173T | x | x | x | x |  |  | M | U | U | U | U |
| G3174T | x | x | x |  |  |  | M | U | U | U | M |
| G3175T | x | x |  |  |  |  | M | U | U | U | U |
| G3176T |  |  |  |  |  |  | M | M | U | U | U |
| G3178T | x | x | x |  |  |  | Failed | U | U | U | U |
| G3179T | x | x | x |  |  |  | M | U | U | U | U |
| G3180T | x | x | x |  |  |  | U | U | U | U | U |
| G3181T | x | x |  |  |  |  | U | U | Failed | U | U |
| G3182T | x | x | x | x | x |  | Failed | U | U | U | U |
| G3183T | x | x | x | x |  |  | M | U | M | M | M |
| G3184T | x | x | x |  |  |  | M | M | U | M | M |
| G3185T | x | x | x | x |  |  | M | U | U | M | U |
| G3186T | x | x | x |  |  |  | M | M | U | U | Failed |
| G3187T | x | x | x | x |  |  | M | M | M | M | M |
| G3189T | x | x | x | x | x |  | M | M | M | M | U |
| G3190T | x | x | x | x |  |  | M | M | M | M | M |
| G3191T | x | x | x |  |  |  | M | M | M | M | U |
| G3192T | x | x | x | x |  |  | M | M | M | M | U |
| G3194T | x | x | x | x |  |  | M | M | U | M | U |
| G3195T | x | x | x | x | x |  | M | M | M | U | U |
| G3196T | x | x | x | x |  |  | M | M | U | U | U |
| G3197T | x | x | x | x | x |  | M | M | M | M | M |
| G3198T | x | x | x | x | x |  | M | M | U | M | U |
| G3199T | x | x | x | x |  |  | M | M | M | M | U |
| G3200T | x | x | x | x | x |  | M | M | U | U | U |
| G3201T | x | x | x |  |  |  | M | U | U | U | Failed |
| G3202T | x | x | x | x | x |  | M | M | M | M | U |
| G3204T | x | x | x | x |  |  | M | M | M | M | M |
| G3205T | x | x |  |  |  |  | M | U | U | M | U |
| G3206T | x | x | x | x | x |  | M | M | M | U | M |
| G3207T | x | x | x |  |  |  | M | Failed | M | U | U |
| G3208T | x | x | x | x | x |  | M | M | M | M | U |
| G3209T | x | x | x | x |  |  | M | M | M | U | M |
| G3210T | x | x | x | x | x |  | M | M | U | M | U |
| G3211T | x | x | x | x |  |  | M | M | M | U | U |
| G3212T | x | x | x | x |  |  | M | M | M | U | U |
| G3213T | x | x | x |  |  |  | M | M | U | M | Failed |
| G3214T |  |  |  |  |  |  | M | M | U | U | U |
| G3215T |  |  |  |  |  |  | U | U | U | M | U |
| G3216T | x | x |  |  |  |  | M | U | U | U | U |
| G3217T | x | x | x | x | x |  | M | M | M | U | U |
| G3218T | x | x | x | x |  |  | M | U | U | U | U |
| G3220T | x | x | x | x |  |  | M | U | U | M | M |
| G3221T | x | x | x | x |  |  | Failed | M | U | U | U |
| G3222T | x | x | x | x |  |  | M | M | M | M | U |
| G3223T | x | x | x | x |  |  | M | M | U | U | U |
| G3224T | x | x | x | x | x |  | M | M | M | U | U |
| G3225T | x | x | x | x | x |  | M | M | U | U | U |
| G3226T | x | x | x |  |  |  | U | U | U | U | U |
| G3227T | x | x | x | x |  |  | M | U | U | U | U |
| G3228T |  |  |  |  |  |  | M | U | U | M | U |
| G3229T | x | x | x | x |  |  | M | M | M | M | U |
| G3231T | x | x | x | x |  |  | M | M | U | M | U |
| G3232T | x | x | x | x |  |  | M | U | M | U | U |
| G3233T | x | x |  |  |  |  | M | U | U | M | U |
| G3235T | x | x | x |  |  |  | M | M | M | M | M |
| G3236T | x | x | x |  |  |  | M | U | U | M | U |
| G3237T | x | x | x | x | x |  | M | M | M | U | M |
| G3238T | x | x | x | x | x |  | M | M | M | U | M |
| G3239T | x | x |  |  |  |  | M | U | M | M | M |
| G3240T | x | x | x | x | x |  | M | M | M | U | M |
| G3241T | x | x | x | x | x |  | M | M | Failed | M | M |
| G3242T | x | x | x | x | x |  | M | M | M | U | M |
| G3243T | x | x | x | x |  |  | M | M | U | U | U |
| G3244T |  |  |  |  |  |  | M | M | M | M | M |
| G3247T | x | x | x | x |  |  | M | U | U | M | U |
| G3249T | x | x |  |  |  |  | U | U | Failed | U | U |
| G3250T | x | x | x | x | x |  | M | M | U | M | U |
| G3251T | x | x | x | x |  |  | M | M | M | M | M |
| G3252T | x | x | x | x | x |  | M | U | M | M | M |
| G3253T | x | x |  |  |  |  | M | U | M | M | U |
| G3254T | x | x | x | x |  |  | M | U | U | M | U |
| G3255T | x | x | x | x |  |  | M | M | U | M | M |
| G3256T | x | x | x | x | x |  | M | M | M | M | M |
| G3257T | x | x | x | x |  |  | M | M | U | M | M |
| G3258T | x | x | x | x |  |  | M | U | U | U | U |
| G3259N | x | x |  |  |  |  | M | U | M | U | U |
| G3261N |  |  |  |  |  |  | U | U | U | U | U |
| G3262T | x | x | x |  |  |  | M | M | M | M | M |
| G3263T | x | x | x | x | x |  | M | M | M | U | U |
| G3264N | x | x | x | x | x |  | M | U | M | U | U |
| G3265N | x | x | x | x | x |  | U | U | U | U | U |
| G3266T | x | x | x | x |  |  | M | M | U | U | U |
| G3270T | x | x | x |  |  |  | U | U | U | U | U |
| G3271T | x | x | x | x | x |  | M | U | M | M | M |
| G3272T | x | x | x | x |  |  | U | U | U | U | U |
| G3274T | x | x | x | x |  |  | M | U | U | U | U |
| G3276T | x | x | x | x | x |  | U | U | M | U | U |
| G3277T | x | x | x | x | x |  | M | M | M | M | M |
| G3279N | x | x | x | x |  |  | M | U | U | U | U |
| G3280T | x | x | x | x | x |  | M | M | M | U | U |
| Results show no correlation between the DNA integrity and the frequency of methylation for any of the final model DNA methylation biomarkers (Pearson correlation coefficient *r* values: *NEFH* 0.214, *GREM1* 0.226, *GATA5* 0.116, *NEURL* 0.040, LAD1 0.019) and between the DNA integrity and the success rate of each gene (Pearson correlation coefficient *r* values: *NEFH* 0.171, *GREM1* 0.022, *GATA5* 0.101, *NEURL* 0.062, *LAD1* 0.087) | | | | | | | | | | | |

SUPPLEMENTARY FIGURES

A


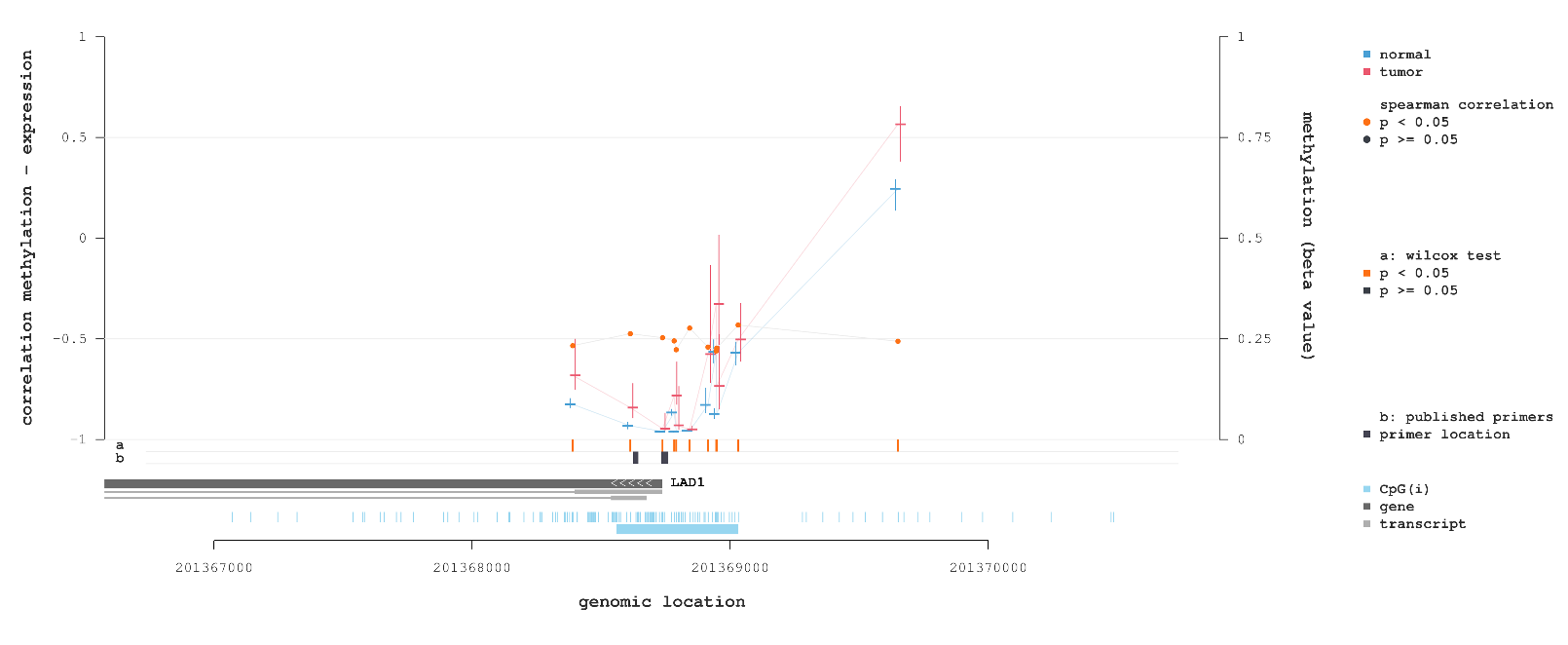

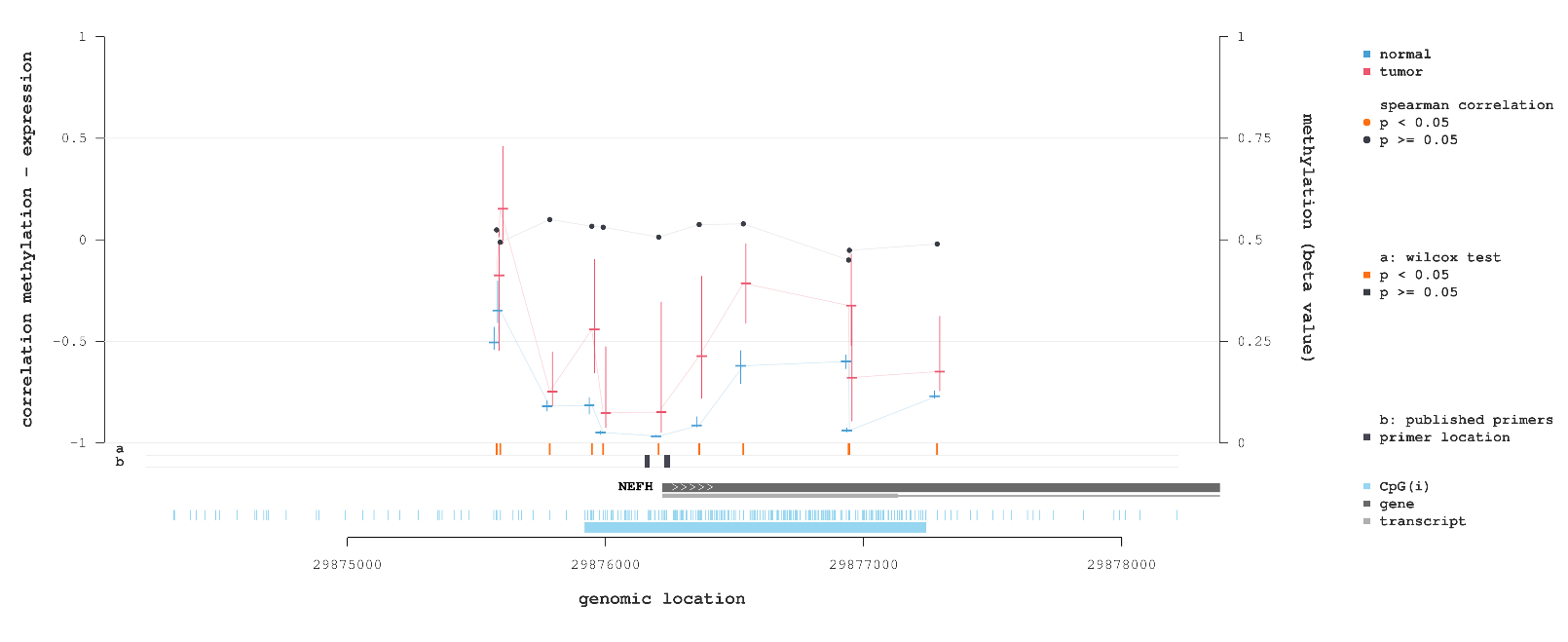

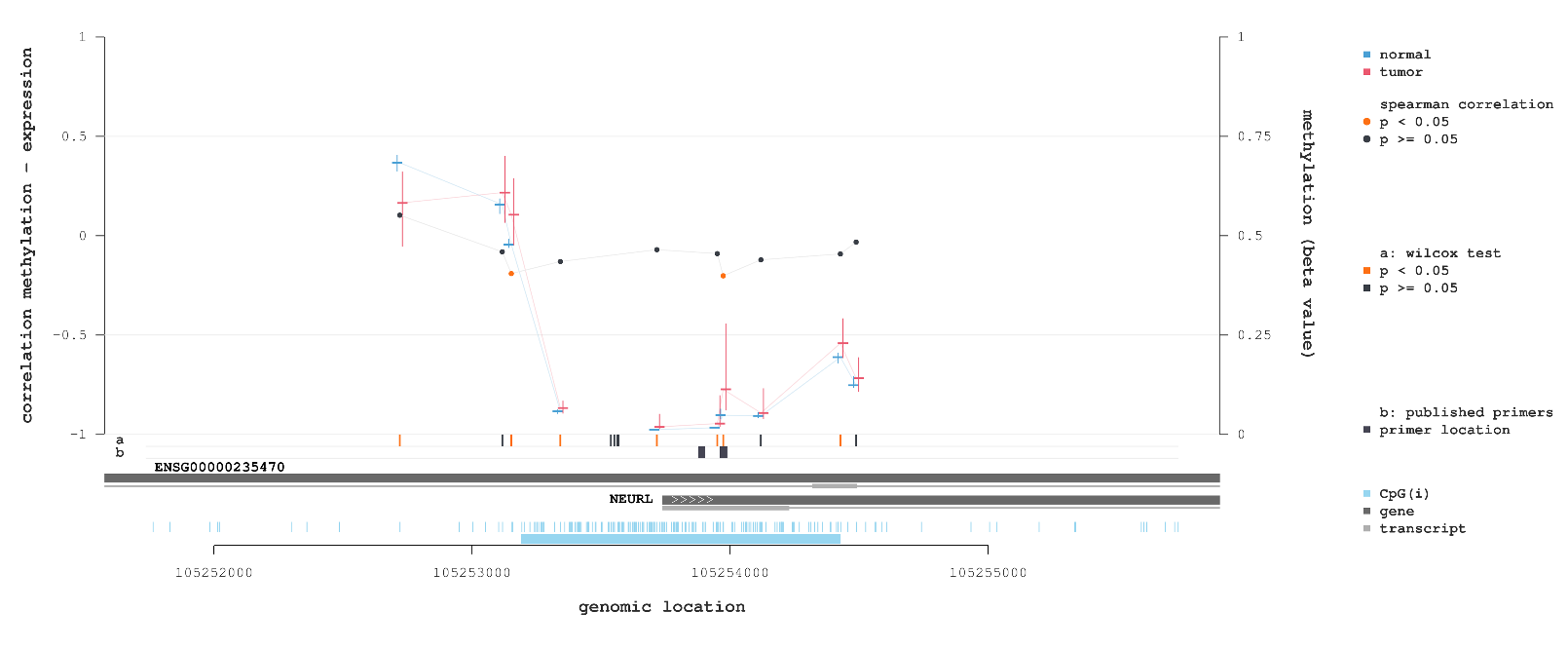

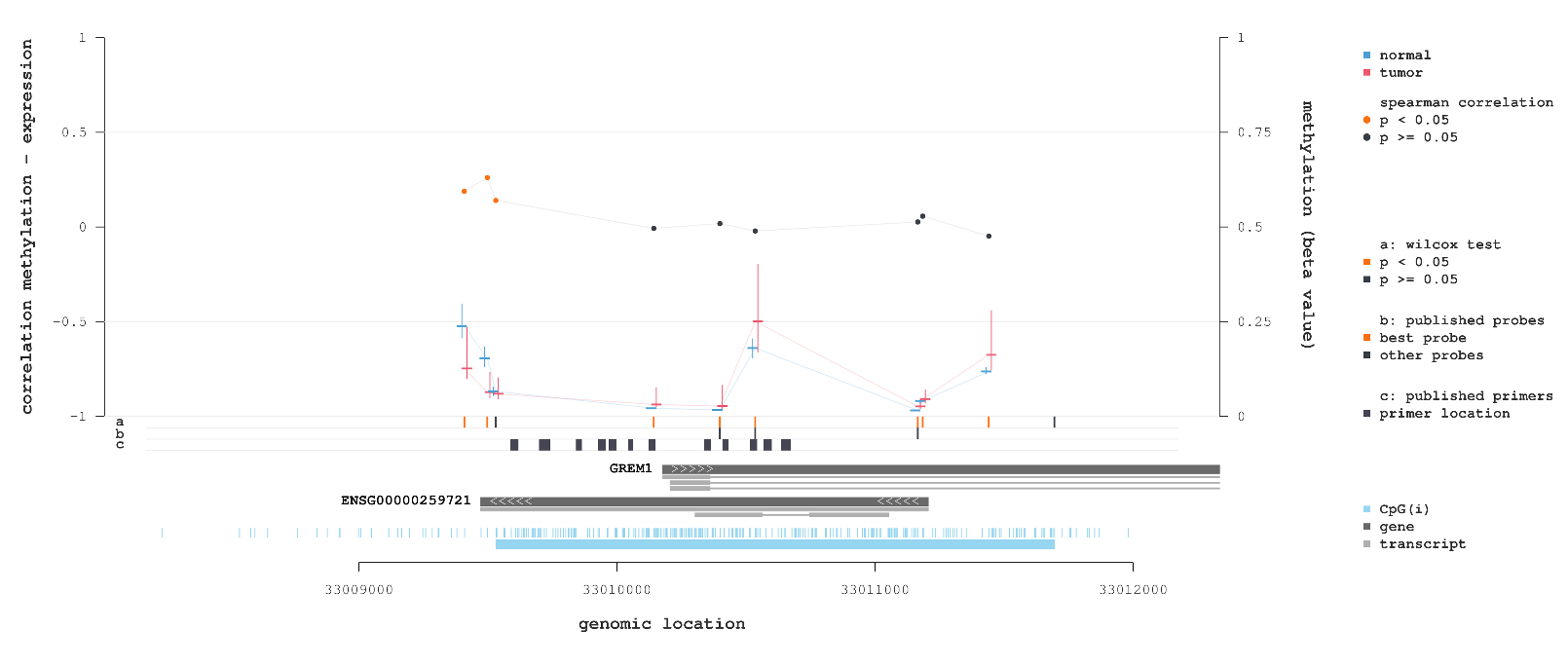

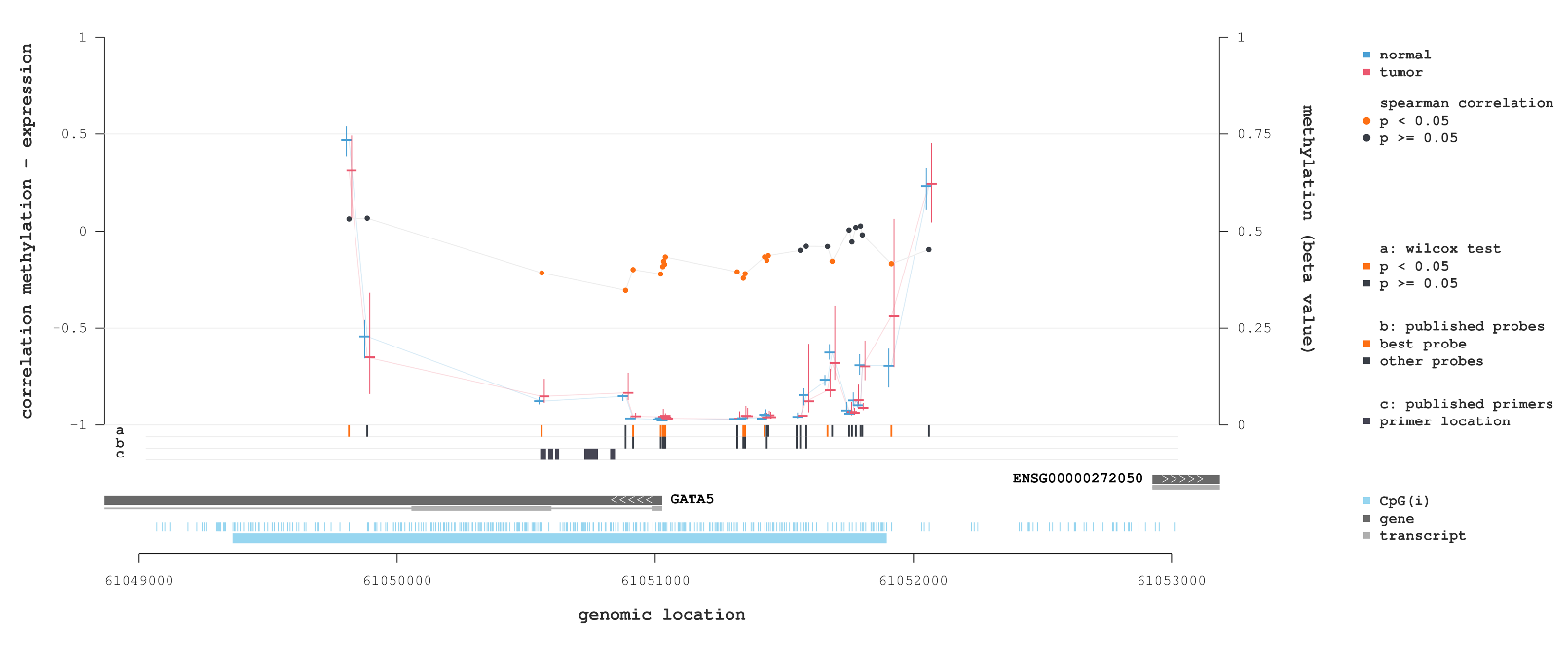


C

B

E

D

**SUPPL FIG S1 | Examples of plots showing the methylation pattern of the promoter region of *LAD1* (A), *NEFH* (B) , *NEURL* (C) ,GREM (D) and *GATA* (E) constructed using publicly available methylation data from TCGA** Summary of the DNA methylation profile around the promoter region in normal (blue) and primary tumor (red) samples. Horizontal lines at each probe location indicate the median beta values for normal and tumor samples; the vertical lines mark the interquartile range. The dots at all probe locations indicate the correlation between the methylation level of the probe and its corresponding gene (Spearman correlation coefficient, orange = p < 0.05, black = p ≥ 0.05). The three tracks (a, b, c) directly under the line chart show the following: a) which probes are differentially methylated between normal and tumor samples (Wilcoxon rank sum test, orange = p < 0.05, black = p ≥ 0.05), b) which probes have been mentioned in the scientific literature, c) the genomic location of DNA methylation-specific primers described in literature. Shown under these three tracks are the locations of any genes and their transcripts within the plotted window, as well as the location of individual CpG dinucleotides and CpG islands.


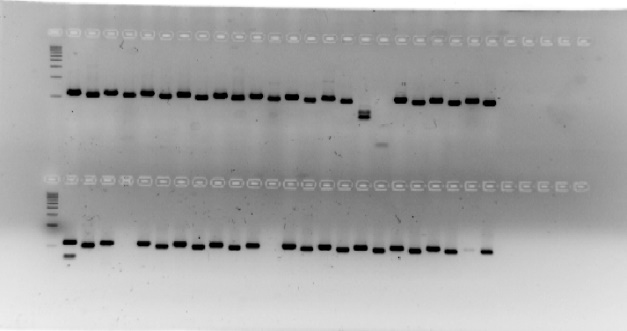

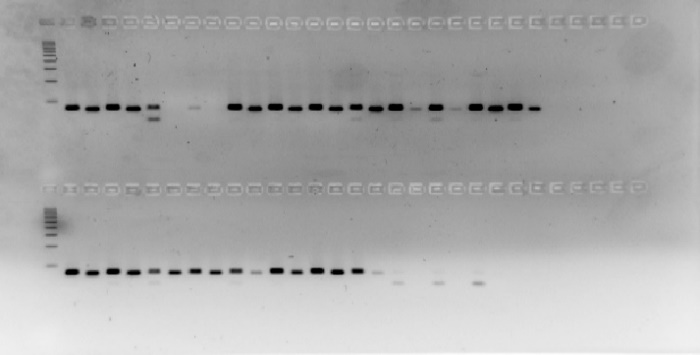

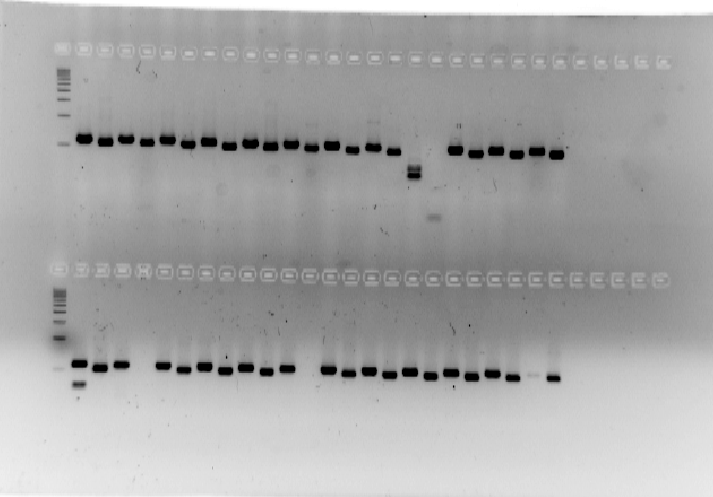

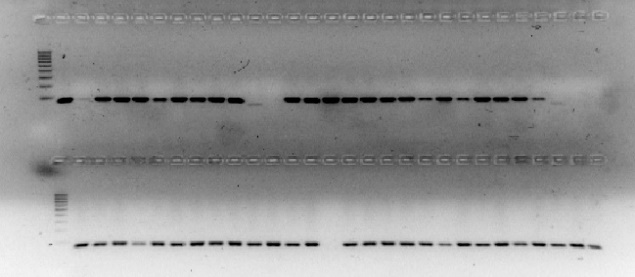

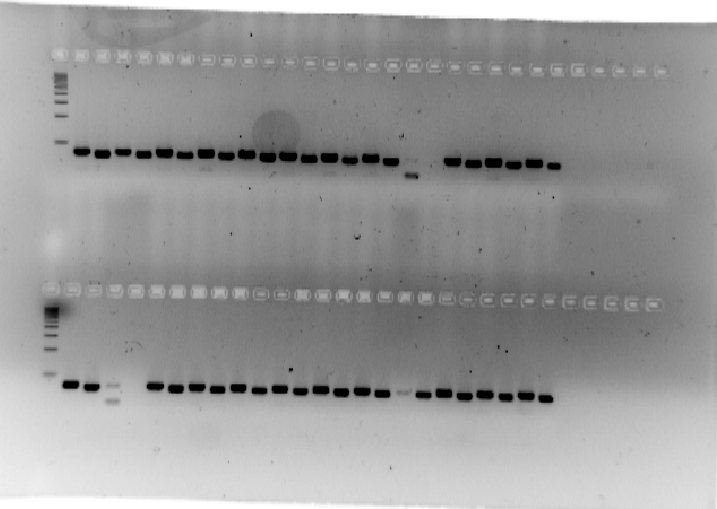

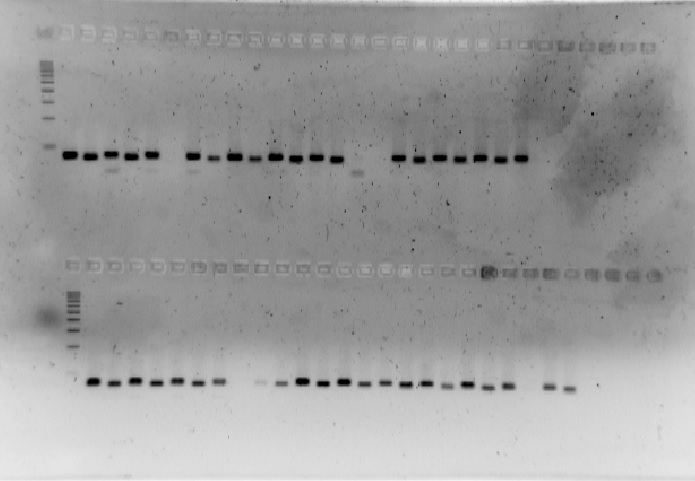


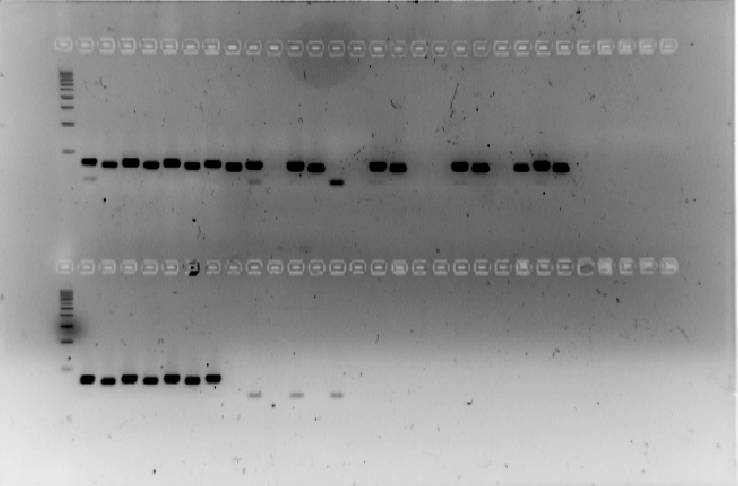


U

M

W1

W2

W3


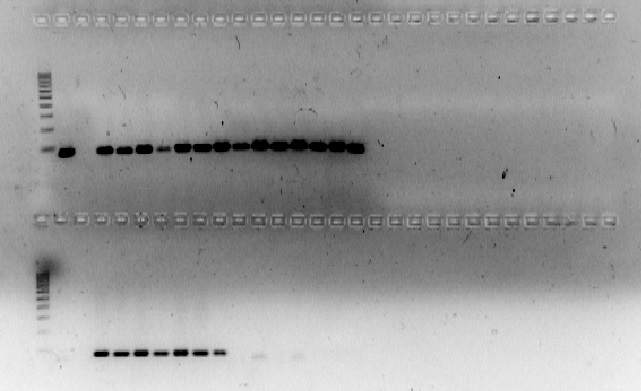


U

M

W1

W2

W3

U

M

W1

W2

W3

6

5

4

3

2

1

b

a

b

a

b

a

b

a

a

b


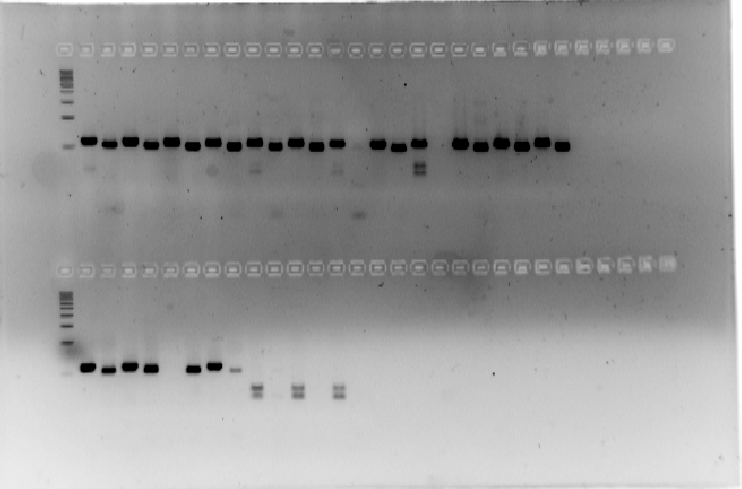


U

M

W1

W2

W3


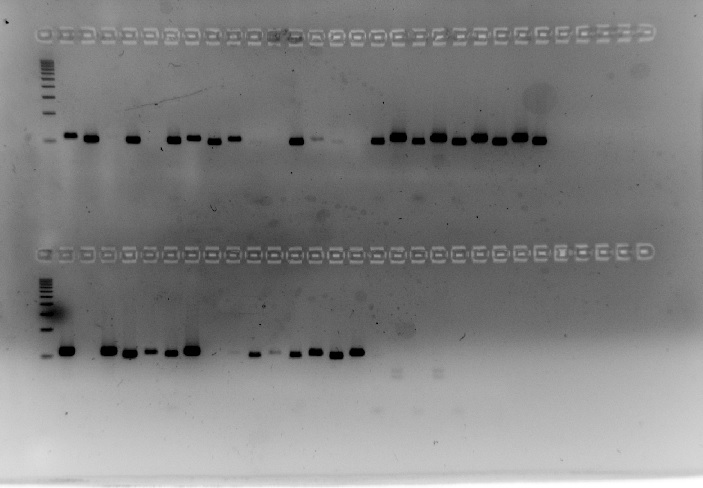


U

M

W1

W2

W3


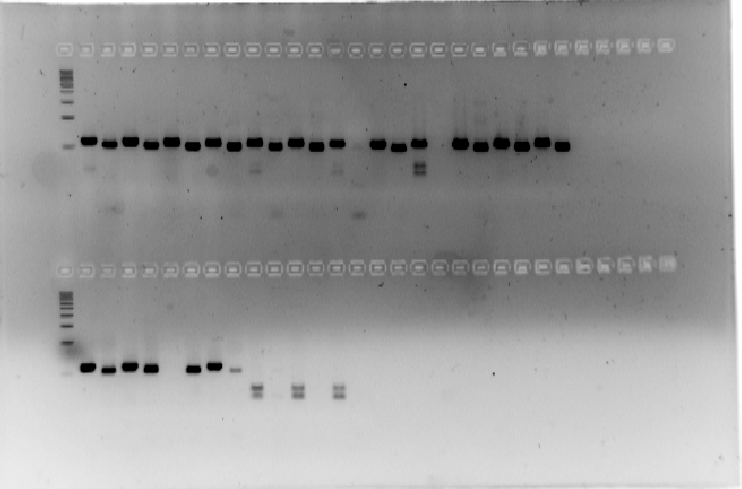


U

M

W1

W2

W3


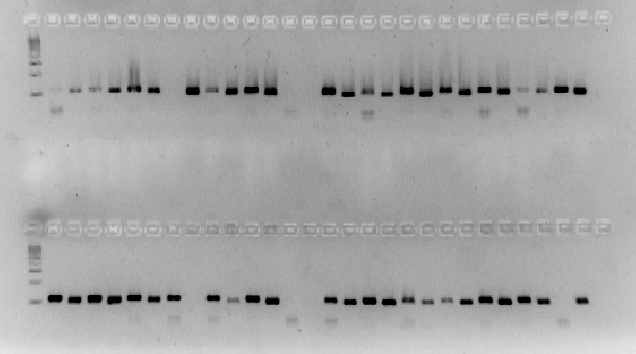

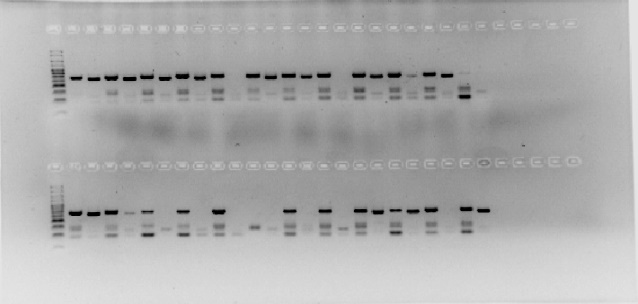

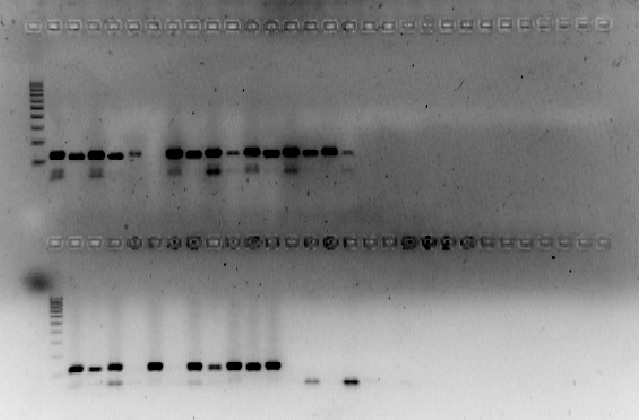

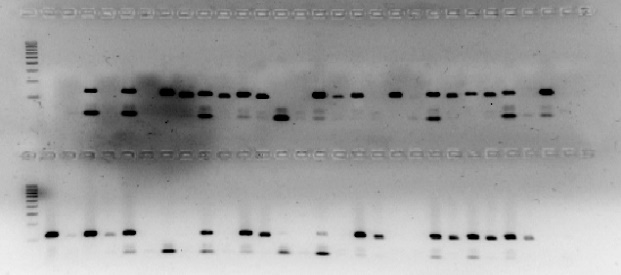

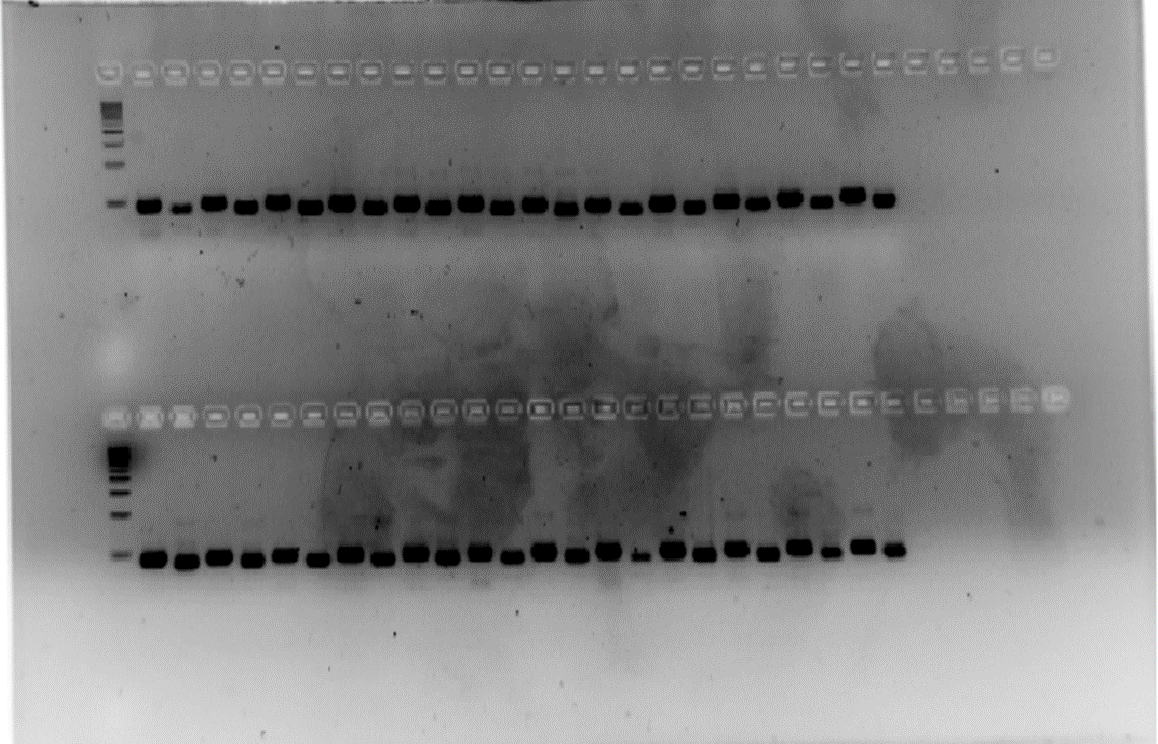

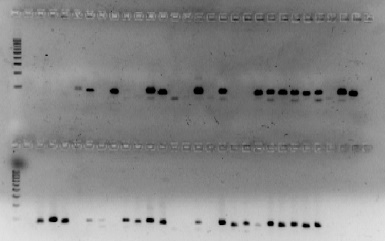

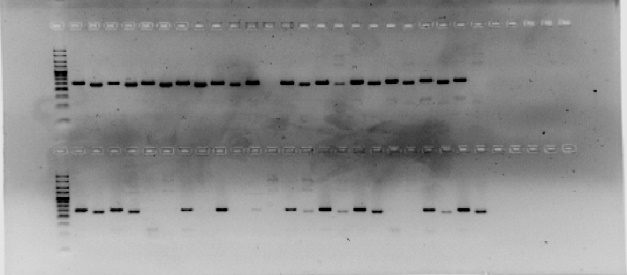

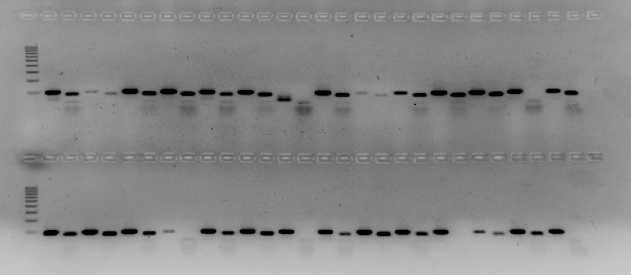


U

M

W1

W2

W3


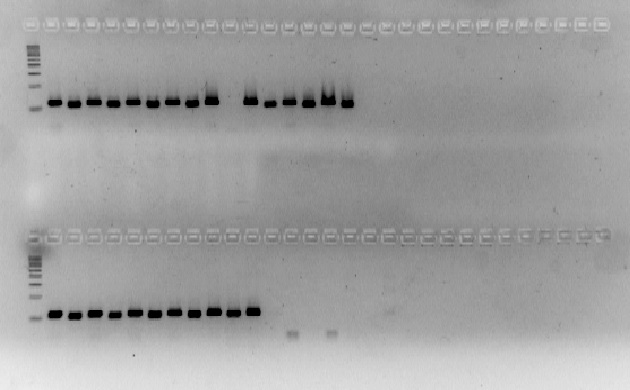


U

M

W1

W2

W3

1310

1210

1110

1010

9

8

7

b

a

b

a

a

b

b

a

a

b

b

a

b

a

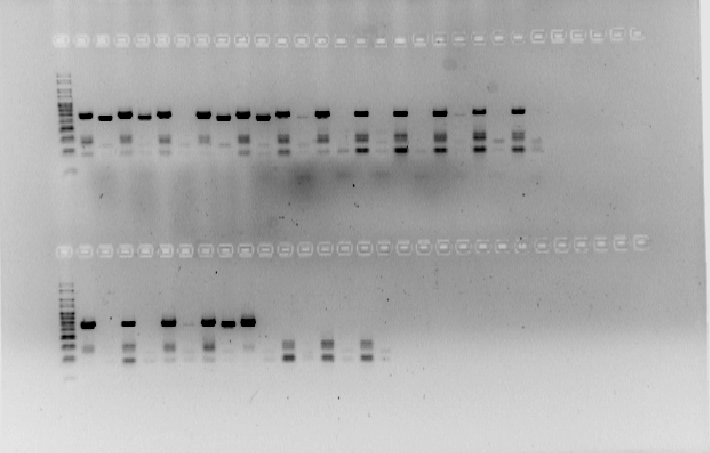


U

M

W1

W2

W3


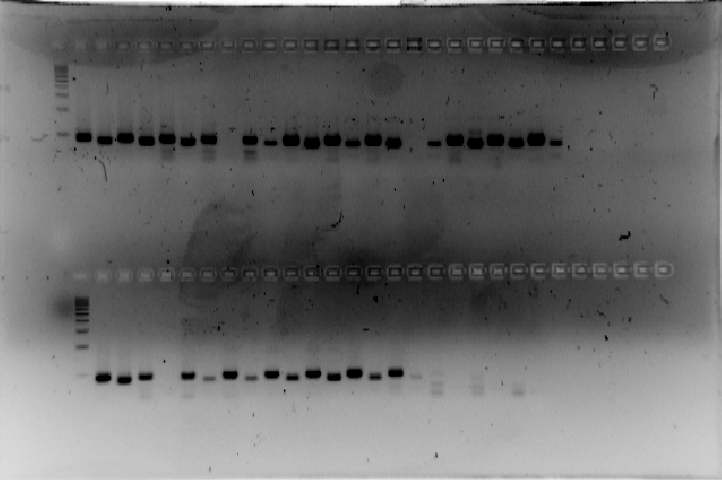


U

M

W1

W2

W3


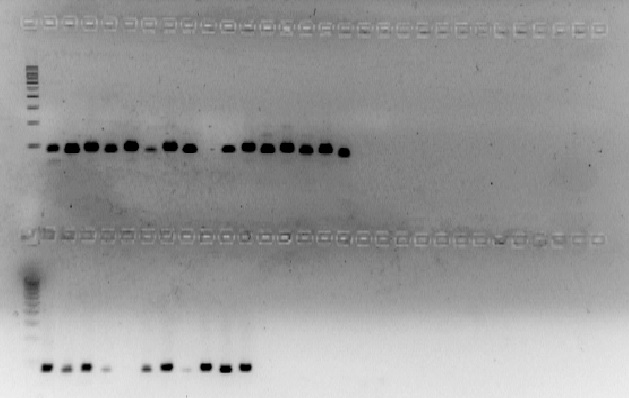


U

M

W1

W2

W3


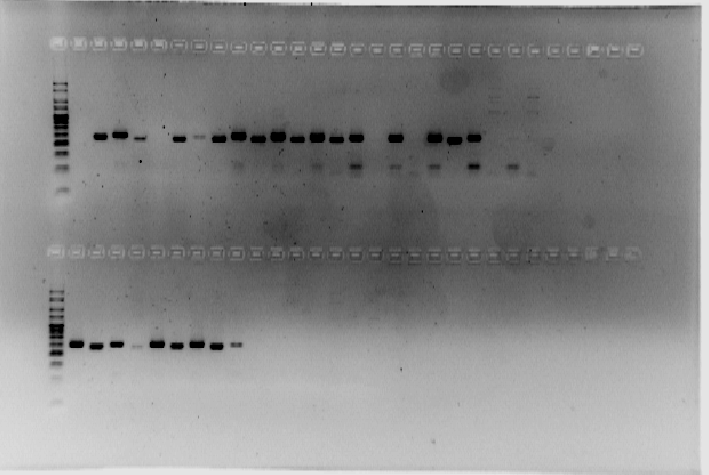


U

M

W1

W2

W3


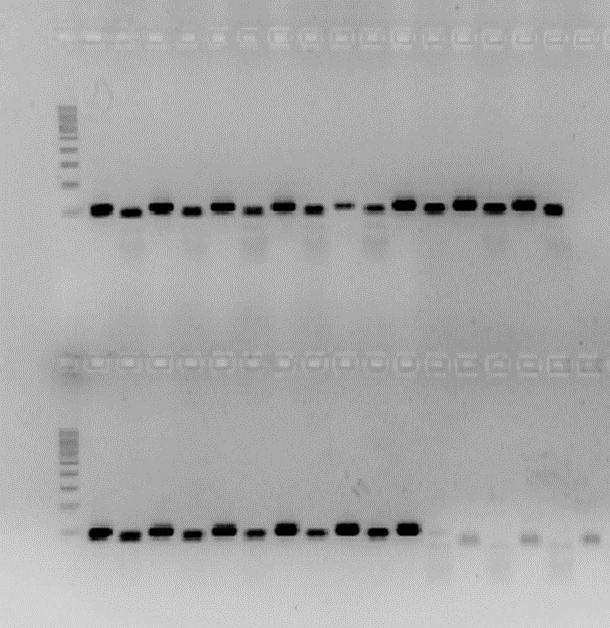


U

M

W1

W2

W3


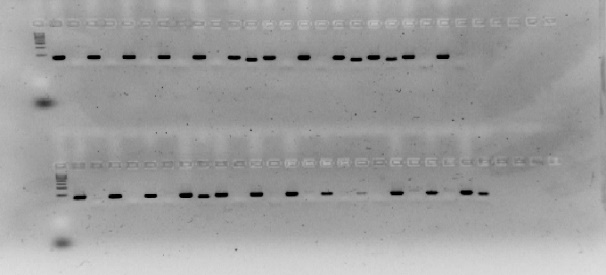

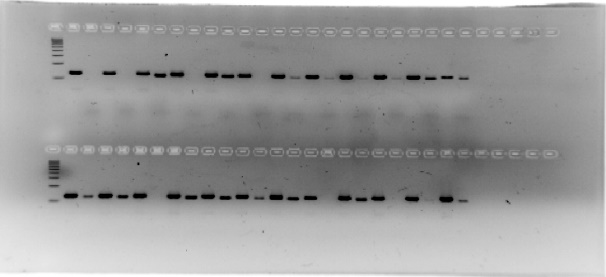

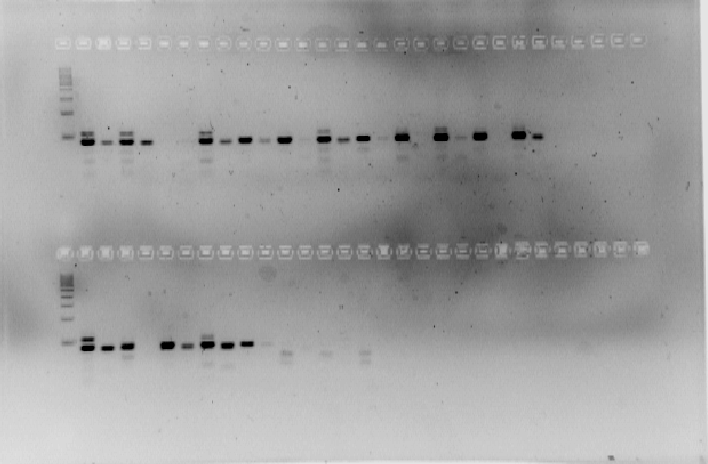

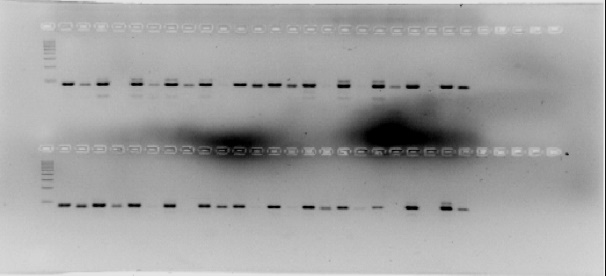

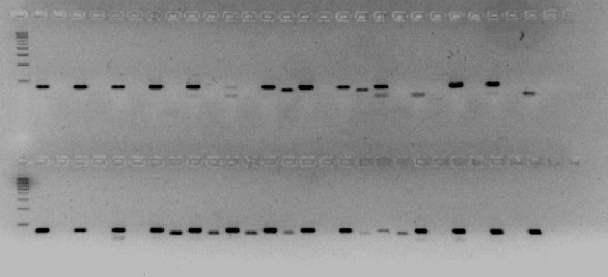

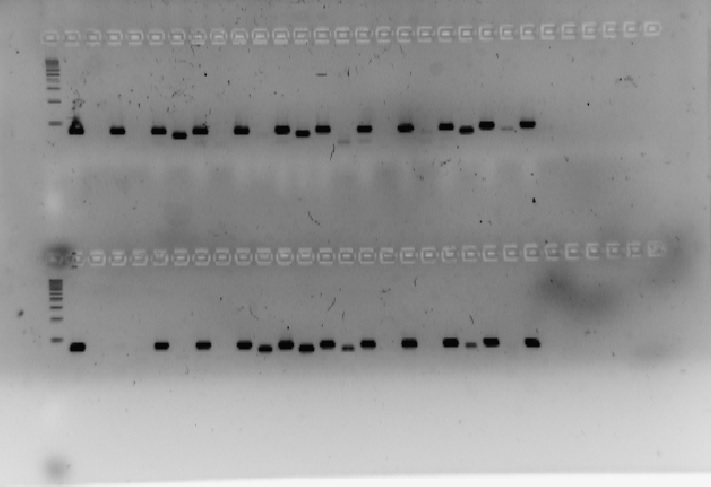

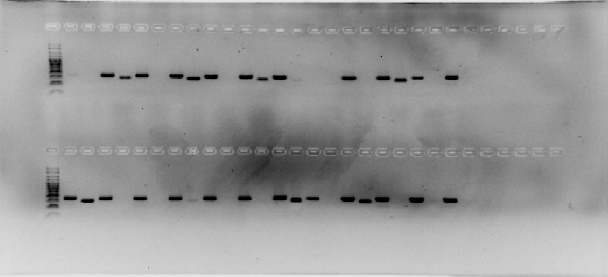

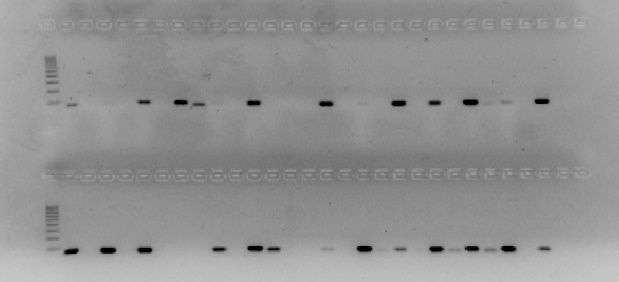


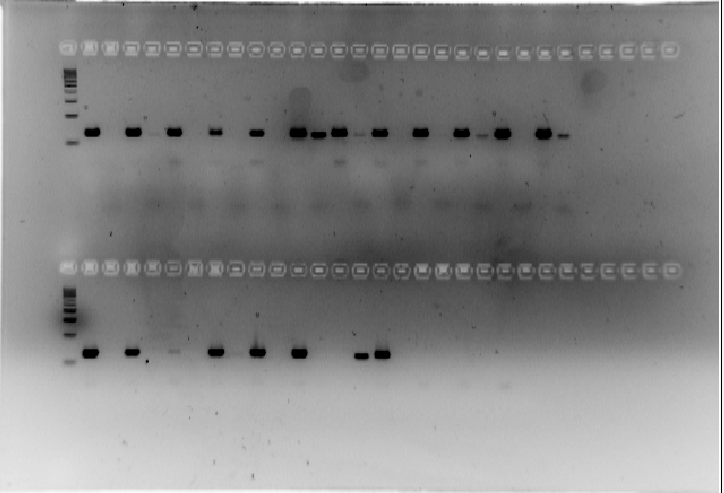


U

M

W1

W2

W3

U

M

W1

W2

W3


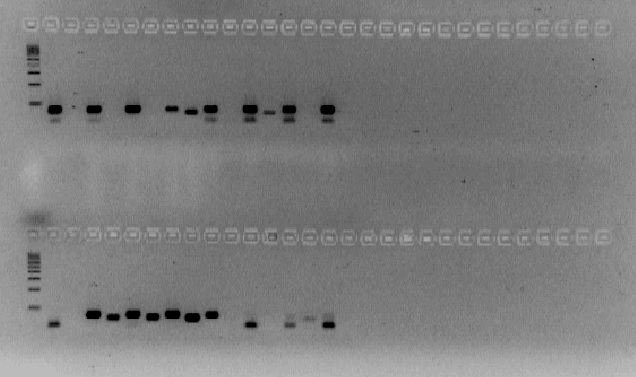


U

M

W1

W2

W3


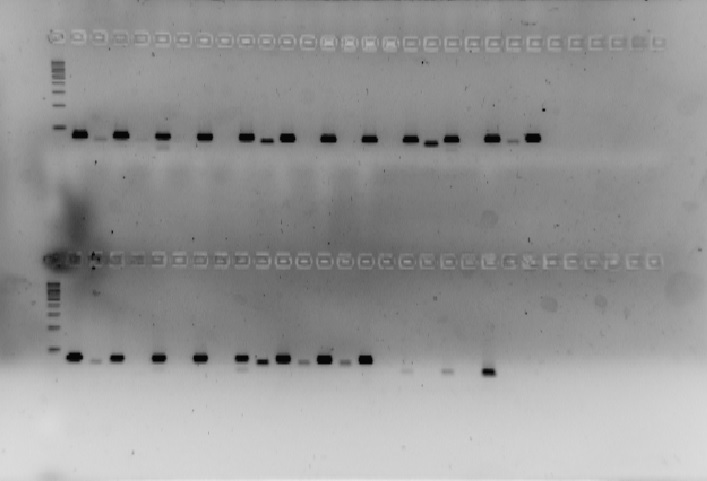


U

M

W1

W2

W3


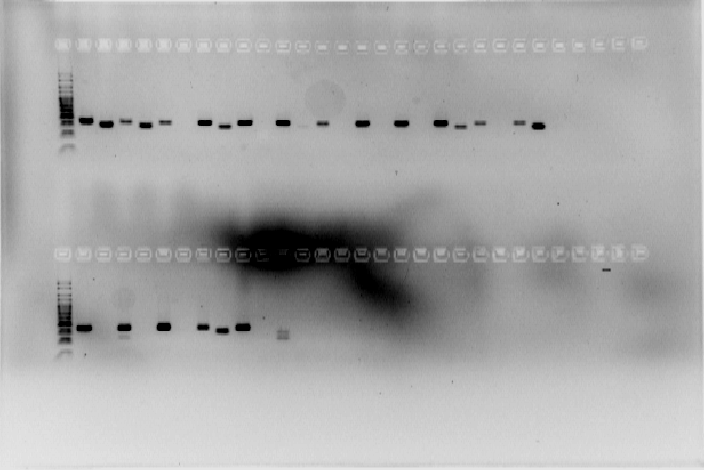


U

M

W1

W2

W3


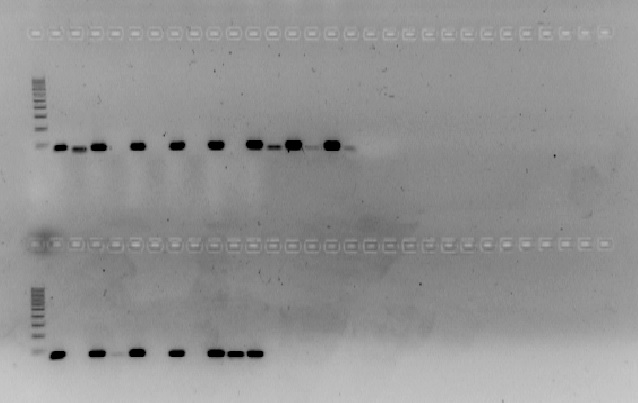


U

M

W1

W2

W3

1910

20

1810

1710

1610

1510

1410

b

a

a

b

b

a

b

a

b

a

b

a

a

b


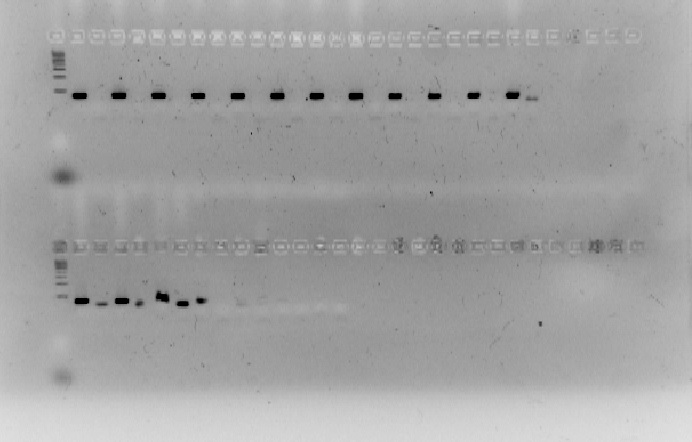


U

M

W1

W2

W3


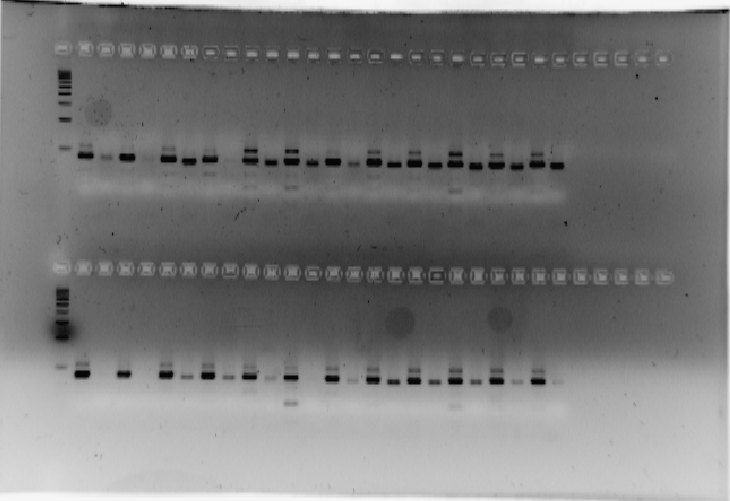

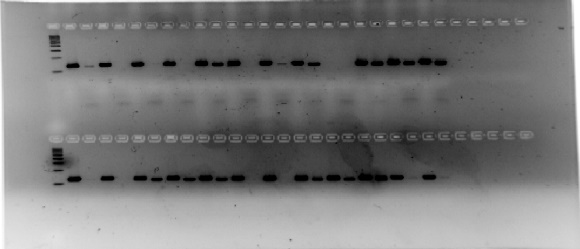


22

**SUPPL FIG S2 | Representative examples of gels obtained from MSP PCR experiments performed on the hospital-based series.** (1, 2 & 3) show examples of *RASSF1A*, (4, 5 & 6) examples of *SCUBE3* (7, 8 & 9) examples of *PCDH8*, (10 & 11) examples of *NEFH,* (12 & 13) examples of BNC1, (14 & 15) examples of *GATA5*, (16, 17 & 18) examples of *LAD1,* (19, 20 & 21) examples of *GREM1*. a & b indicate that the two gels’ pictures belong to the same PCR run. Every two adjacent gel lanes belong to the same sample with the first well for the Unmethylated reaction and the second well for the methylated reaction. Methylated samples should show a band in the methylated reaction lane ,independent whether the unmethylated lane shows a band or not, with the band intensity being at least one third of the control methylated band. Unmethylated samples should show only a band in the unmethylated reaction lane. The “U” control should only show a band only in the unmethylated lane, the “M” control should show a band in the methylated reaction lane and/or a band in the unmethylated reaction lane. The water control from the bisulfite reaction (W1), the outside PCR (W2) and the inside PCR (W3) should show no band with the exclusion of primers dimers. Samples that show no amplification in any of the unmethylated or methylated wells should be reported as failed and be repeated. PCR gels were run in combination with a 100bp ladder for 20 minutes at 150 V.

21

b

a

a

b


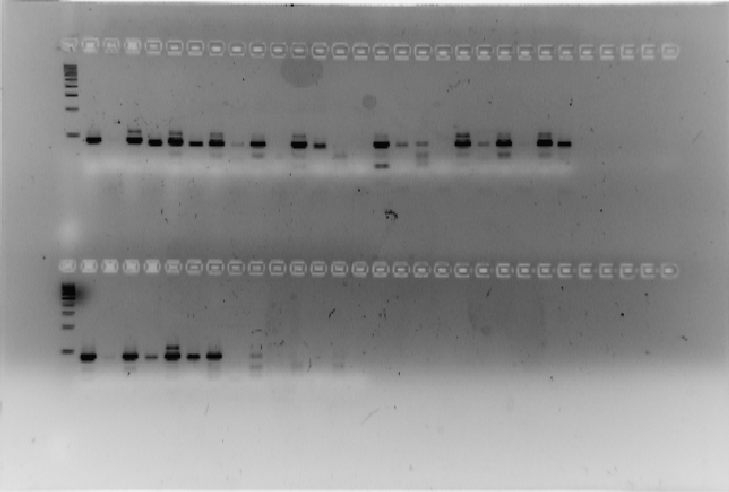


U

M

W1

W2

W3


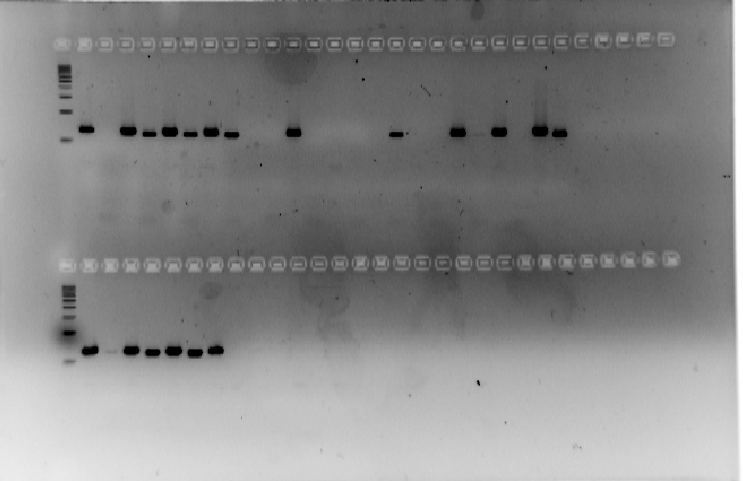


U

M

W1

W2

W3


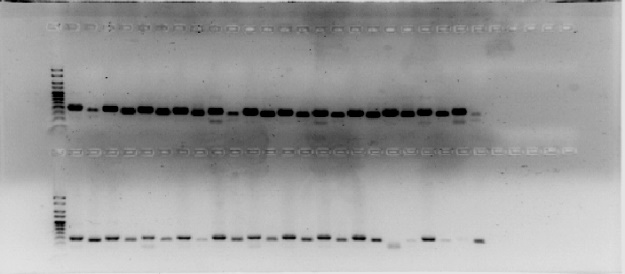

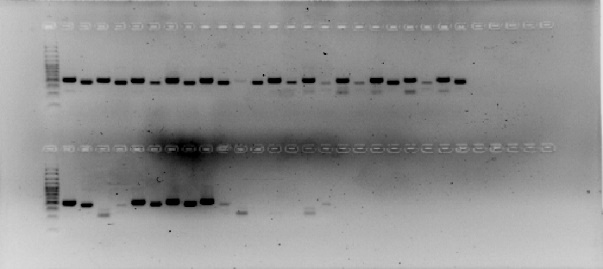

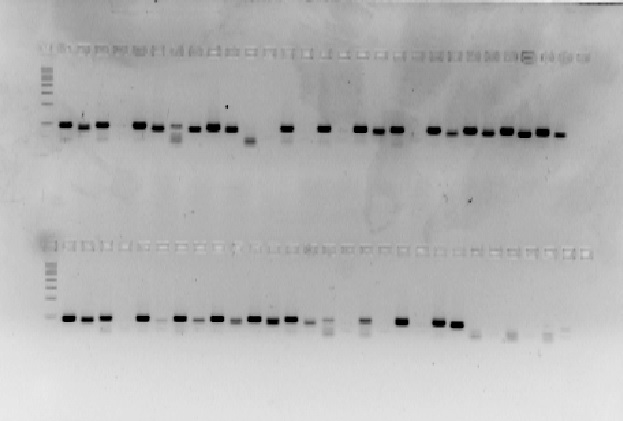


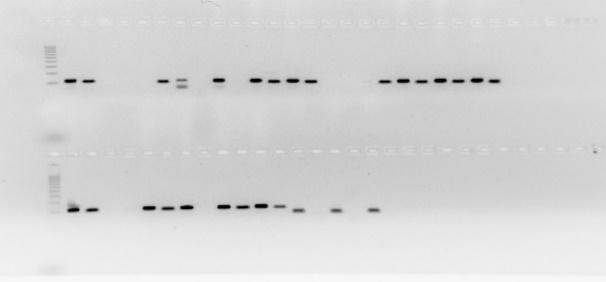


U

M

W1

W2

W3

3c

3b

3a


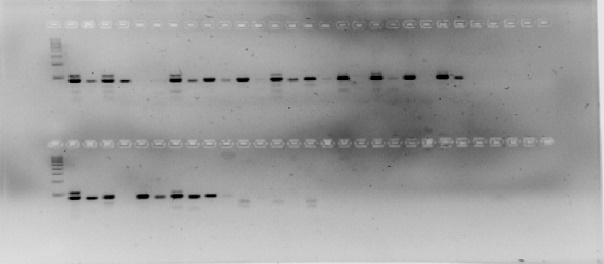


U

M

W1

W2

W3

2c

2b

2a

1c

1b

1a


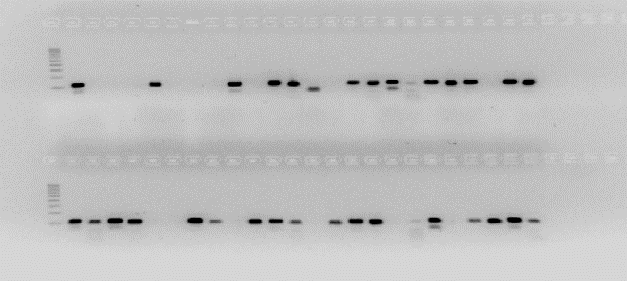


U

M

W1

W2

W3


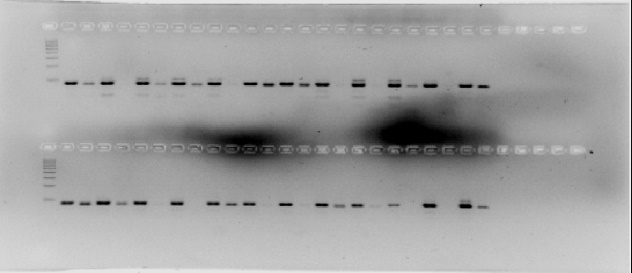


M

U

W1

W2

W3


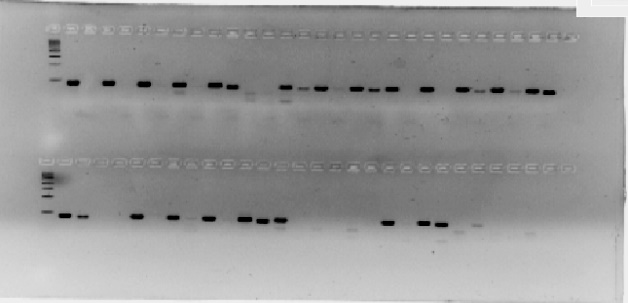


M

U

W1

W2

W3


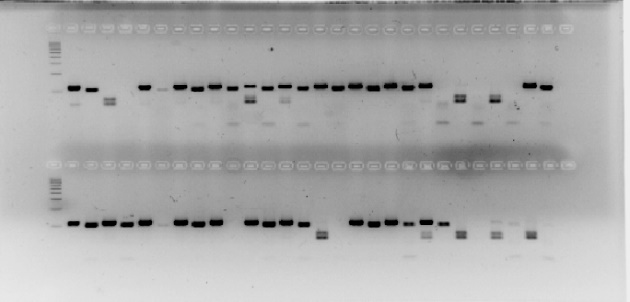


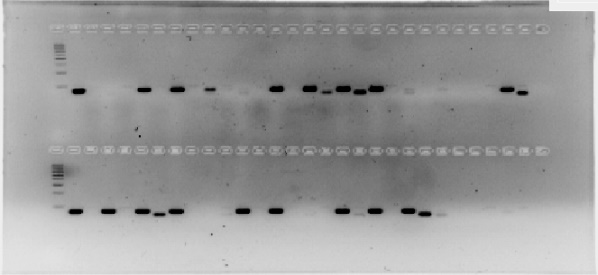

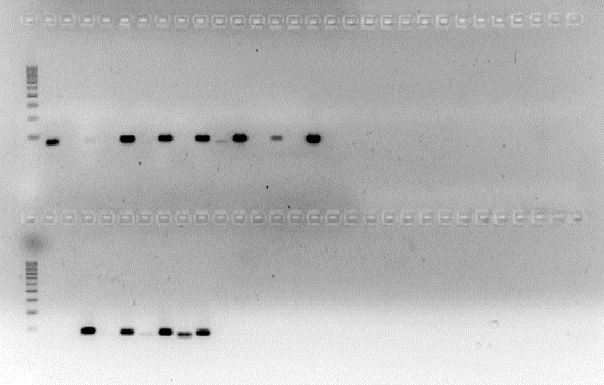

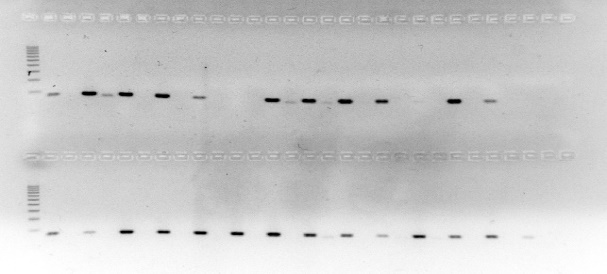

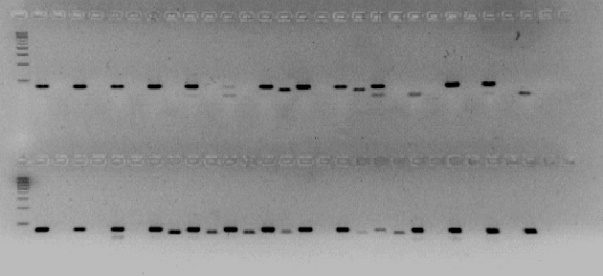


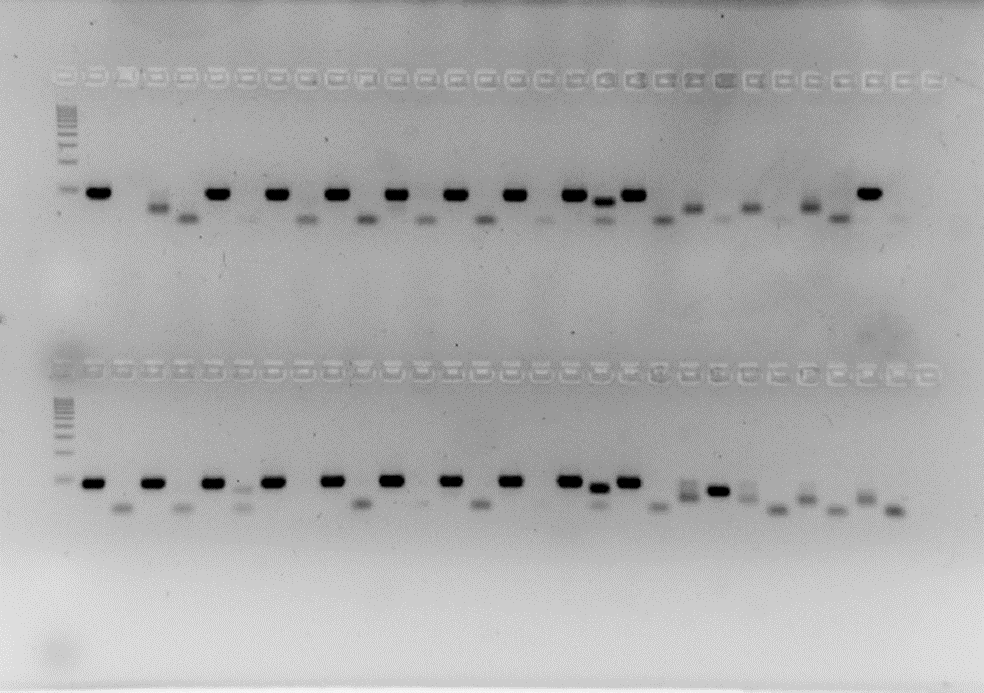

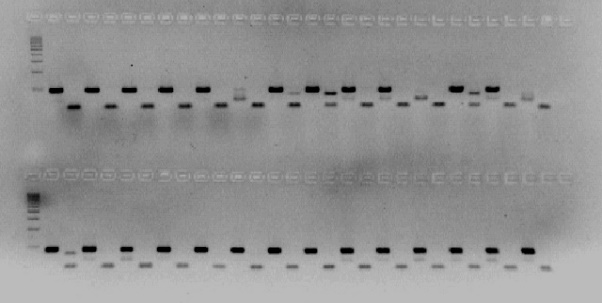


**SUPPL FIG S3 | Representative examples of gels obtained from MSP PCR reproducibility experiments performed on the population-based series** (1a) represents a reproducibility MSP PCR gel for *SCUBE3,* (1b) & (1c) show the original PCR gels for the indicated samples. (2a) represents a reproducibility MSP PCR gel for *GREM2* (2b) &, (2c) show the original PCR gels for the indicated samples. (3a) represents a reproducibility MSP PCR gel for *NEFH2*, (3b) &, (3c) show the original PCR gels for the indicated samples. (4a) represents a reproducibility MSP PCR gel for *LAD1*, (4b) & (4c) show the original PCR gels for the indicated samples. (5a) represents a reproducibility MSP PCR gel for *NEURL2*, (5b) & (5c) show the original PCR gels for the indicated samples. (6a) represents a reproducibility MSP PCR gel for *GATA5*, (6b) & (6c) show the original PCR gels for the indicated samples. Samples are indicated in blue and red to show performance in the reproducibility and original PCR experiments.

6c

6b

6a

5c

5b

5a

4c

4b

4a


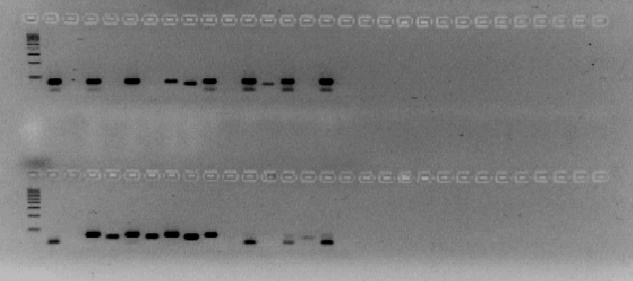


U

M

W1

W2

W3

U

W2

W3

W1

M


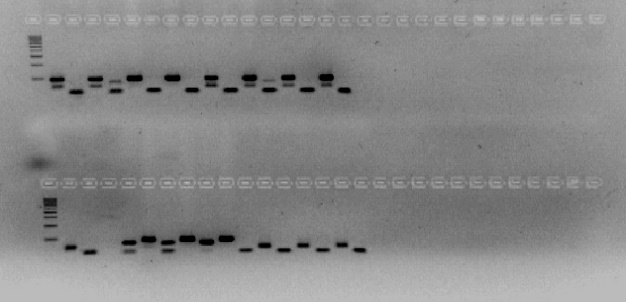


U

M

W1

W2

W3


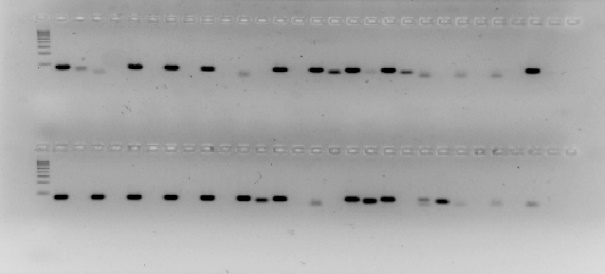


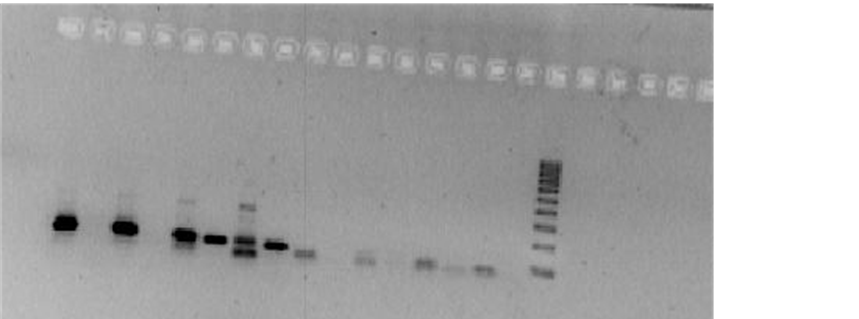


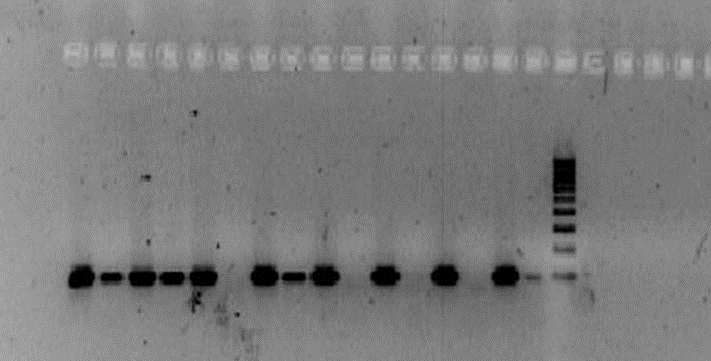


1

U

U

M

M

W1

W2

W3

W3

b

a


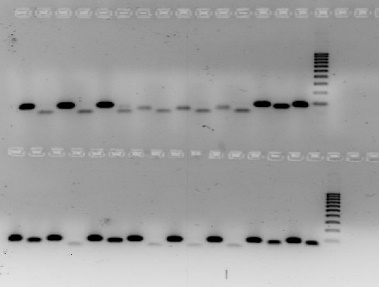

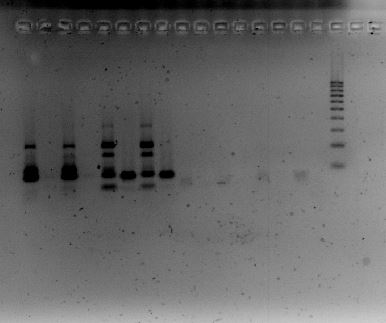

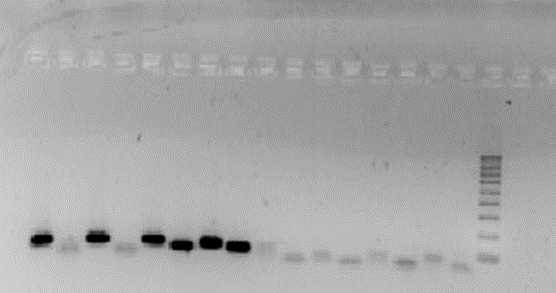

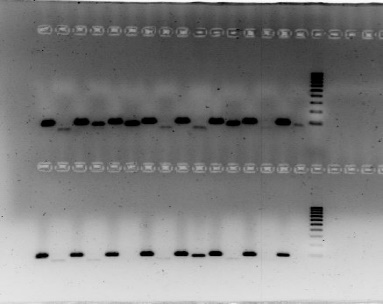

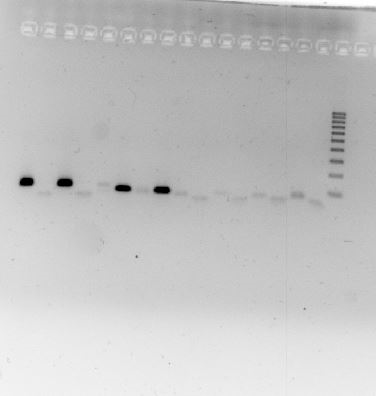

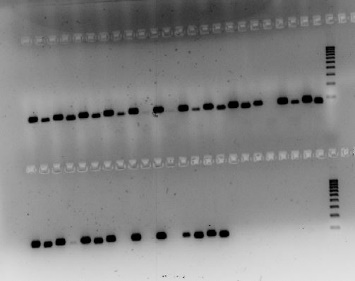

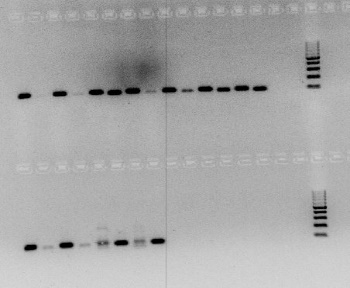

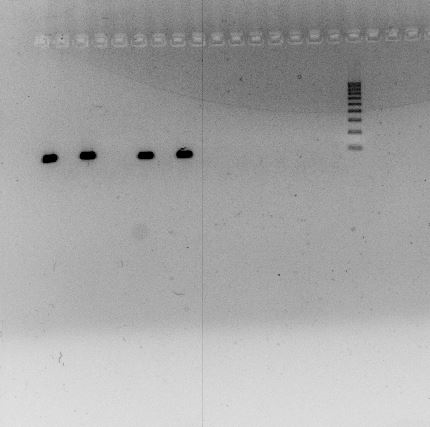

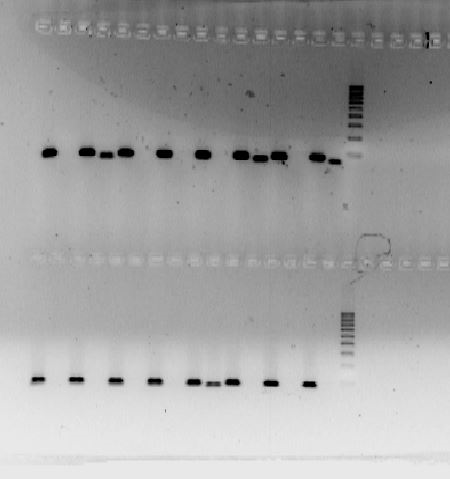

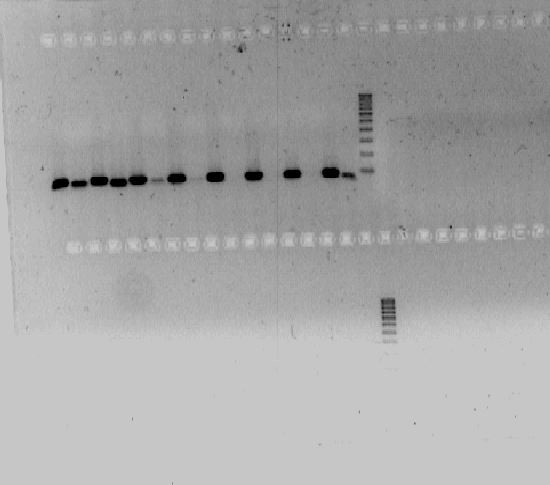

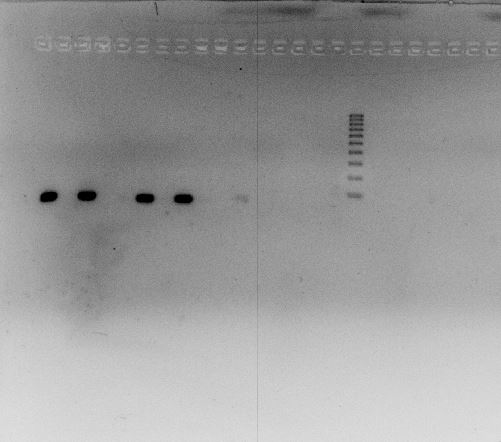

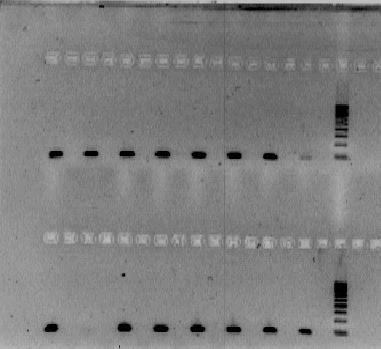


U

U

M

M

W1

W2

W3

W3

U

U

M

M

W1

W2

W3

W3

U

U

M

M

W1

W2

W3

W3

U

U

M

M

W1

W2

W3

W3

a

b

b

a

U

U

M

M

W1

W2

W3

W3

U

U

M

M

W1

W2

W3

W3

U

U

M

M

W1

W2

W3

W3

b

a

b

a

b

a

6

7

5

4

2

3


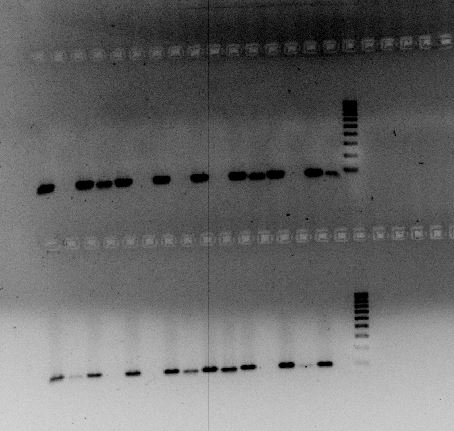

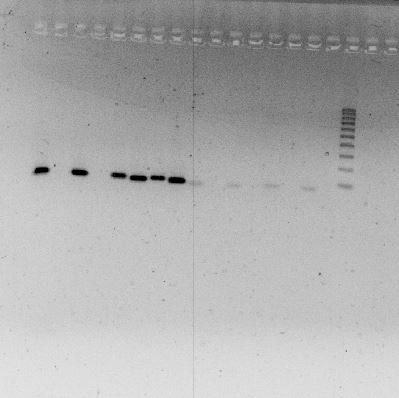


U

U

M

M

W1

W2

W3

W3

8

**SUPPL FIG S4 | Representative examples of gels obtained from MSP PCR experiments performed on the hospital-based series.** (1 & 2) show examples of *GREM1*, (3 & 4) examples of *GATA5*, (5 & 6) examples of *NEFH*, (7 & 8) examples of *NEURL* and (8 & 9) examples of *LAD1*. a & b indicate that the two gels’ pictures belong to the same PCR run. Every two adjacent gel lanes belong to the same sample with the first well for the Unmethylated reaction and the second well for the methylated reaction. Methylated samples should show a band in the methylated reaction lane, independent whether the unmethylated lane shows a band or not, with the band intensity being at least one third of the control methylated band. Unmethylated samples should show only a band in the unmethylated reaction lane. The “U” control should only show a band only in the unmethylated lane, the “M” control should show a band in the methylated reaction lane and/or a band in the unmethylated reaction lane. The water control from the bisulfite reaction (W1), the outside PCR (W2) and the inside PCR (W3) should show no band with the exclusion of primers dimers. Samples that show no amplification in any of the unmethylated or methylated wells should be reported as failed and be repeated. PCR gels were run in combination with a 100bp ladder for 20 minutes at 150 V.

a

b


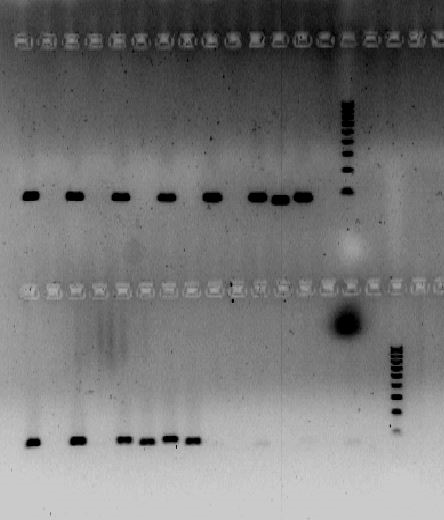


9

U

U

M

M

W1

W2

W3

W3


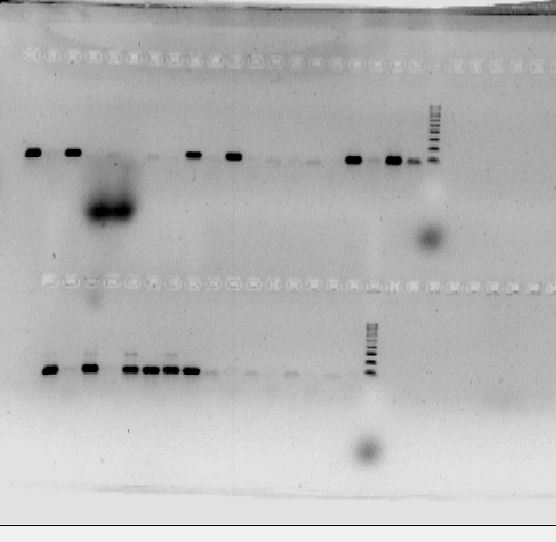


U

U

M

M

W1

W2

W3

W3

1a

**SUPPL FIG S5 | Representative examples of gels obtained from MSP PCR reproducibility experiments performed on the hospital-based series** (1a) represents a reproducibility MSP PCR gel for *GREM1*, (1b) & (1c) show the original PCR gels for the indicated samples. (2a) represents a reproducibility MSP PCR gel for *GATA5*(2b) & (2c) show the original PCR gels for the indicated samples. (3a) represents a reproducibility MSP PCR gel for *LAD1*, (3b) & (3c) show the original PCR gels for the indicated samples. Samples are indicated in blue and red to show performance in the reproducibility and original PCR experiments.


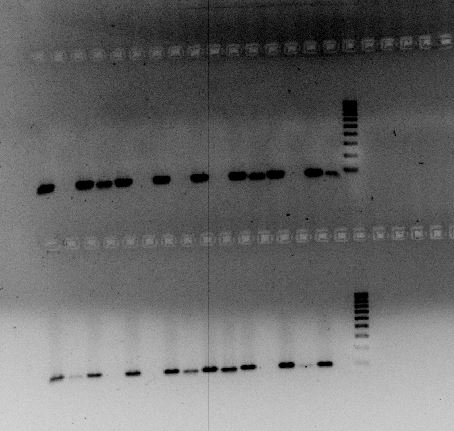

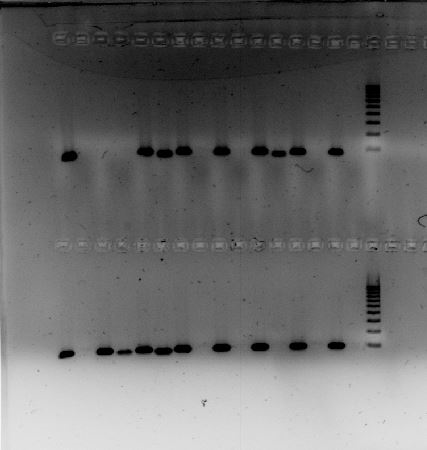

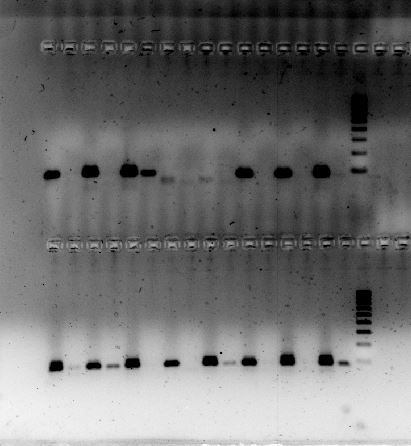


3c

3b

3a

2c

2b

2a

1c

1b


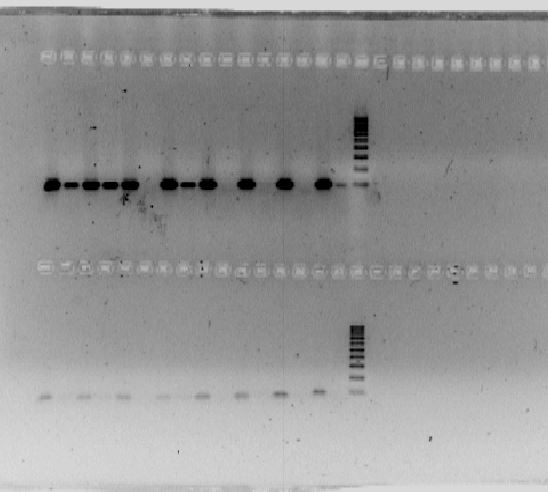

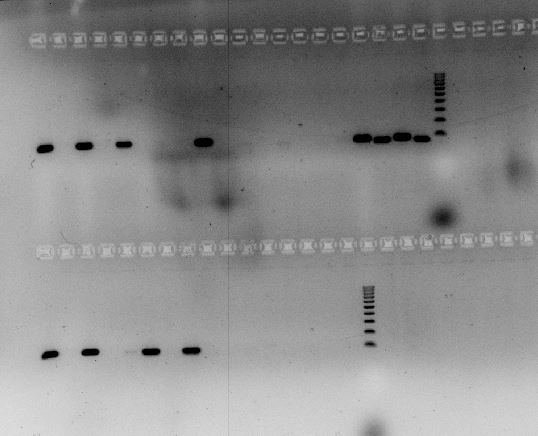


U

U

M

M

W1

W2

W3

W3


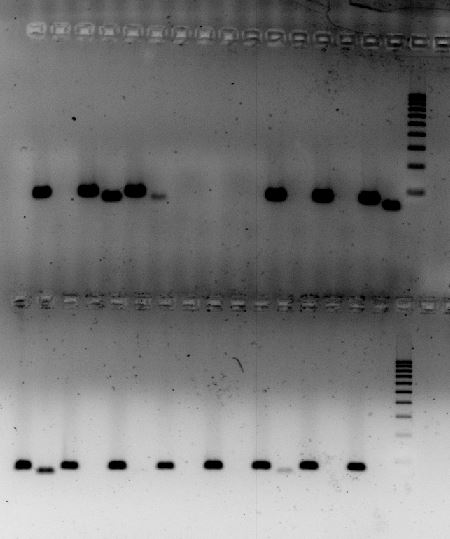

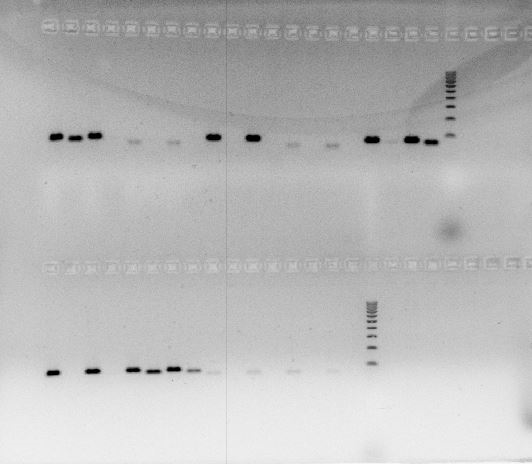


U

U

M

M

W1

W2

W3

W3


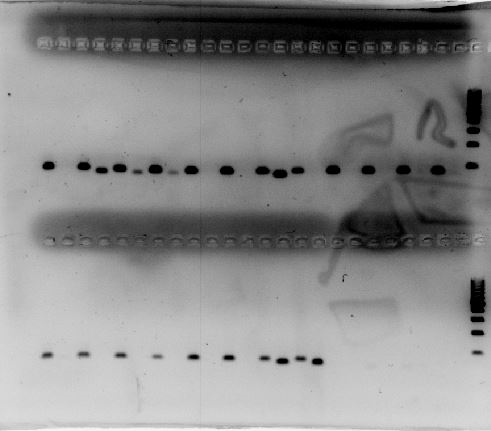


U

U

M

W1

W2

W3

W3

M


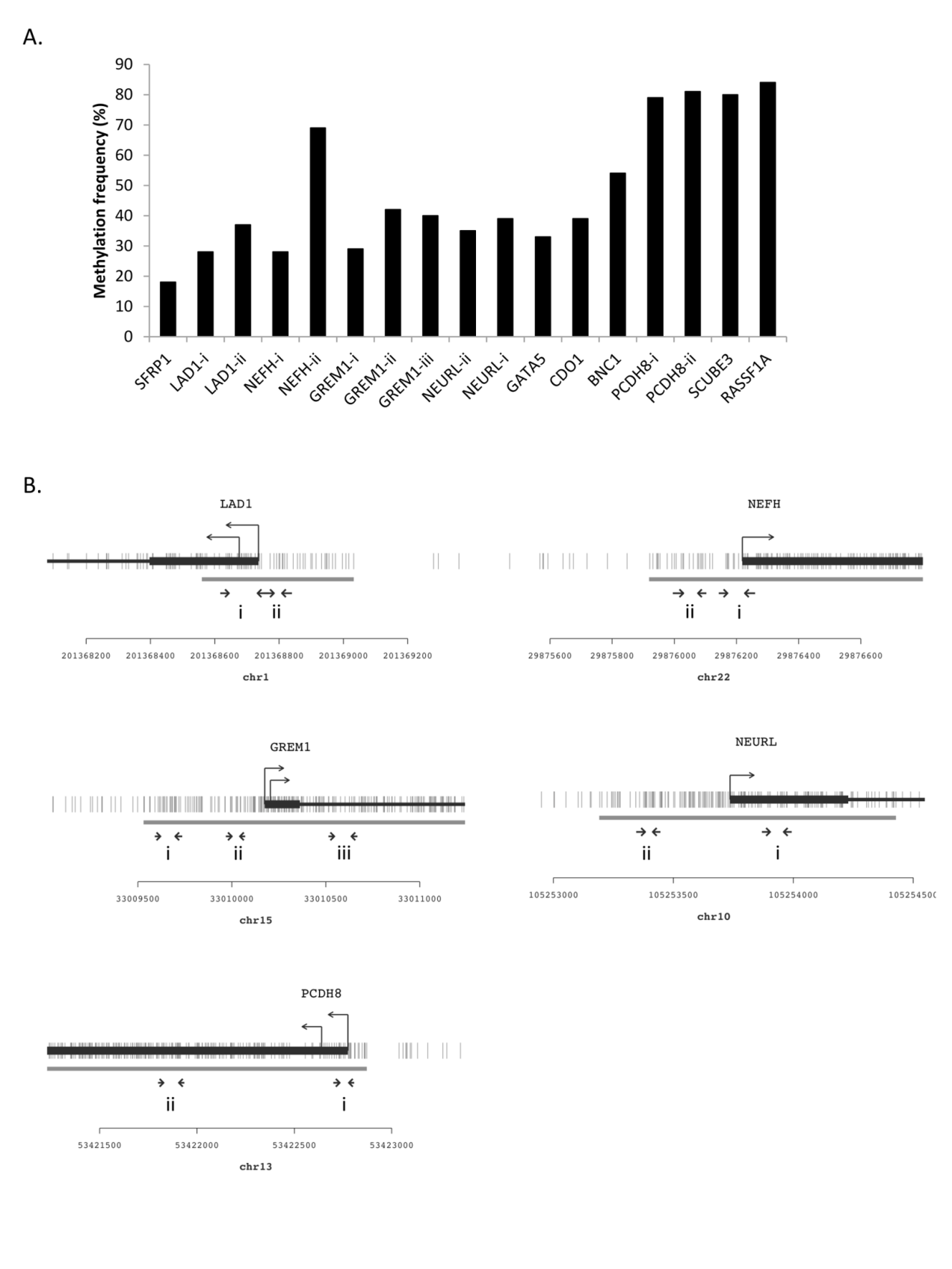


**SUPPL FIG S6 | Epigenetic characterization of ccRCCs.** A. Promoter methylation frequency of the studied genes (including the different genomic locations within a gene promoter). B. Visual representation of the promoter region of the genes for which more than one MSP assay was developed, including the location of the different primer sets with horizontal arrows.

**SUPPL FIG S7 | Overall cause-specific survival curves for candidate methylation markers in the population-based ccRCC series.** Kaplan-Meier curves of *GREM1-i* (A) HR 1.92, CI 1.25-2.95, *GATA5* (B) HR 1.68, CI 1.11-2.54, *LAD1-i* (C) HR 2.25, CI 1.51-3.35, *LAD1-ii* (D) HR 1.90, CI 1.28-2.82, *NEURL-ii* (E) HR 1.99, CI 1.30-3.03, *NEFH-i* (F) HR 1.76, CI 1.16-2.66, and *SFRP1* (G) HR 2.23, CI 1.43-3.49 analyzed for overall cause-specific survival. *P*-value estimated by log-rank test, HR: hazard ratio, 95%-CI: 95%-confidence interval. U: unmethylated; M: methylated

p=0.0022

A

B

p=0.0117

E

C

G

D

F

p<0.0001

p=0.0013

p=0.0010

p=0.0067

p=0.0002

p=0.2012

B

p=0.0536

C

p=0.8800

D

p=0.1133

E

p=0.1846

F

p=0.7972

G

H

p=0.1533

p=0.9680

p=0.3640

I

J

A

p=0.5662

p=0.9680

I

p=0.3640

J

p=0.1533

H

**SUPPL FIG S8 | Overall cause-specific survival curves for candidate methylation markers in the population-based ccRCC series.** Kaplan-Meier curves of *GREM1-ii* (A) HR .88, CI 0.59-1.32, *GREM1-iii* (B) HR 1.28, CI 0.87-1.91, *CDO1* (C) HR 1.48, CI 0.98-2.21, *SCUBE3* (D) HR 0.95, CI 0.89-2.70, *PCDH8-i* (E) HR 1.56, CI 0.98-2.21, *PCDH8-ii* (F) HR 0.73, CI 0.46-1.17, *RASSF1A* (G) HR 0.93, CI 0.55-1.57, *NEURL-i* (H) HR 1.32, CI 0.90-1.95, *NEFH-ii* (I) HR 1.00, CI 0.65-1.55, and *BNC1* (J) HR 1.20, CI 0.79-1.81, analyzed for overall cause-specific survival. *P*-value estimated by log-rank test, HR: hazard ratio, 95%-CI: 95%-confidence interval: U: unmethylated; M: methylated
